# Virome Data Explorer: A web resource to longitudinally explore respiratory viral infections, their interactions with other pathogens and host transcriptomic changes in over 100 people

**DOI:** 10.1371/journal.pbio.3002089

**Published:** 2024-01-18

**Authors:** Marta Galanti, Juan Angel Patiño-Galindo, Ioan Filip, Haruka Morita, Angelica Galianese, Mariam Youssef, Devon Comito, Chanel Ligon, Benjamin Lane, Nelsa Matienzo, Sadiat Ibrahim, Eudosie Tagne, Atinuke Shittu, Oliver Elliott, Tomin Perea-Chamblee, Sanjay Natesan, Daniel Scholes Rosenbloom, Jeffrey Shaman, Raul Rabadan

**Affiliations:** 1 Department of Environmental Health Sciences, Mailman School of Public Health, Columbia University, New York, New York, United States of America; 2 Program for Mathematical Genomics, Department of Systems Biology, Columbia University Irving Medical Center, New York, New York, United States of America; New York University School of Medicine, UNITED STATES

## Abstract

Viral respiratory infections are an important public health concern due to their prevalence, transmissibility, and potential to cause serious disease. Disease severity is the product of several factors beyond the presence of the infectious agent, including specific host immune responses, host genetic makeup, and bacterial coinfections. To understand these interactions within natural infections, we designed a longitudinal cohort study actively surveilling respiratory viruses over the course of 19 months (2016 to 2018) in a diverse cohort in New York City. We integrated the molecular characterization of 800+ nasopharyngeal samples with clinical data from 104 participants. Transcriptomic data enabled the identification of respiratory pathogens in nasopharyngeal samples, the characterization of markers of immune response, the identification of signatures associated with symptom severity, individual viruses, and bacterial coinfections. Specific results include a rapid restoration of baseline conditions after infection, significant transcriptomic differences between symptomatic and asymptomatic infections, and qualitatively similar responses across different viruses. We created an interactive computational resource (Virome Data Explorer) to facilitate access to the data and visualization of analytical results.

## Abstract 2

Viral respiratory infections are an important public health concern, due to their prevalence, transmissibility, and potential to cause serious disease. This study describes a rich dataset of host/pathogen data collected in New York City during 2 years of active sampling for endemic respiratory viruses, and uses it to characterize the transcriptomic effect of infections presenting with different symptomatology and etiology.

## Introduction

Respiratory infections are a major cause of disease worldwide, impose enormous societal and public health costs, and are a leading cause of death in young children [1,2]. The recent emergence of Severe Acute Respiratory Syndrome Coronavirus 2 (SARS-CoV-2) has underscored the need for better understanding of the transmissibility, pathogenicity, and host/pathogen interactions of the many different viruses and bacteria that cause respiratory infections. While some viruses like influenza and respiratory syncytial virus (RSV) are more routinely tracked and frequently studied, a comprehensive analysis of host/pathogen interactions is lacking for many of the other common viruses that circulate widely and cause respiratory disease. Understanding the mechanisms of host response is essential for appropriately understanding the diseases caused by these infectious agents, directing surveillance, treating infections, and developing targeted therapeutics against severe disease. Rather than direct viral effects, host response to infection is often the predominant factor determining symptom severity [3].

Transcriptomic analysis provides the opportunity to simultaneously characterize host and respiratory pathogen interactions [4,5]. This approach has been validated using peripheral blood, nasopharyngeal samples [6,7], and lung cell samples [8–10]. Most existing studies focusing on transcriptomic response to respiratory infections consist of either in vitro analyses [8–12], challenge studies in which participants are inoculated with a viral agent in a controlled setting [13–17], or observational studies tracking symptomatic natural infection [6,18–20]. Challenge studies are informative because they can capture both symptomatic and asymptomatic infections and can be conducted in a very controlled manner with sampling of participants at specific and frequent time points. However, induced infections are not necessarily representative of naturally acquired infections and the reduced size of these experiments (typically involving 10 to 15 subjects), the age of the participants (typically avoiding very young and older individuals), and the lack of bacterial coinfections makes them prone to biases that are difficult to control. Observational transcriptomic studies, on the other hand, typically follow cases after participants manifest respiratory symptoms and are thus biased towards symptomatic and severe infections often resulting in hospitalization [6,7,9,21]. This is a major limitation, given that previous analyses have found that the majority of respiratory infections are asymptomatic or mild [22] and predominantly medically unattended [23].

Here, we leverage a unique dataset generated from comprehensive longitudinal surveillance of common respiratory viruses over the course of 19 months in the Manhattan borough of New York City. For our study, we collected epidemiological data, including self-reported respiratory symptoms, from 214 participants. Importantly, we also processed over 4,000 nasal swabs, analyzed corresponding qPCR-based respiratory virus panel assays, and sequenced over 800 samples for host transcriptome characterization and quantification of respiratory viruses, pathogenic bacteria, and fungi. Using these data, we created an interactive web resource, publicly available website *The Virome of Manhattan Project*: *Virome Data Explorer* to visualize longitudinal transcriptomic changes driven by respiratory viruses in naturally occurring infections.

In order to illustrate the utility of our dataset for future research and beyond, here we depict the transcriptomic changes that occurred during infection, accounting for differences in symptomatology, the type of viral infection and the occurrence of coinfection with respiratory bacteria. We find the up-regulation of hundreds of genes functionally associated with immune response during viral infection. Further, we identify a gene expression signature that is not only capable of differentiating between symptomatic and asymptomatic infections, but also between positive asymptomatic individuals and negative individuals (either symptomatic or asymptomatic). We also depict differences between viruses, with influenza leading to greater changes in gene expression than other viruses, such as coronavirus (CoV) or rhinovirus. Finally, we find evidence of interactions, at the transcriptomic level, between common respiratory bacteria and viruses.

## Results

### The Virome of Manhattan Project data collection

**Fig 1:**
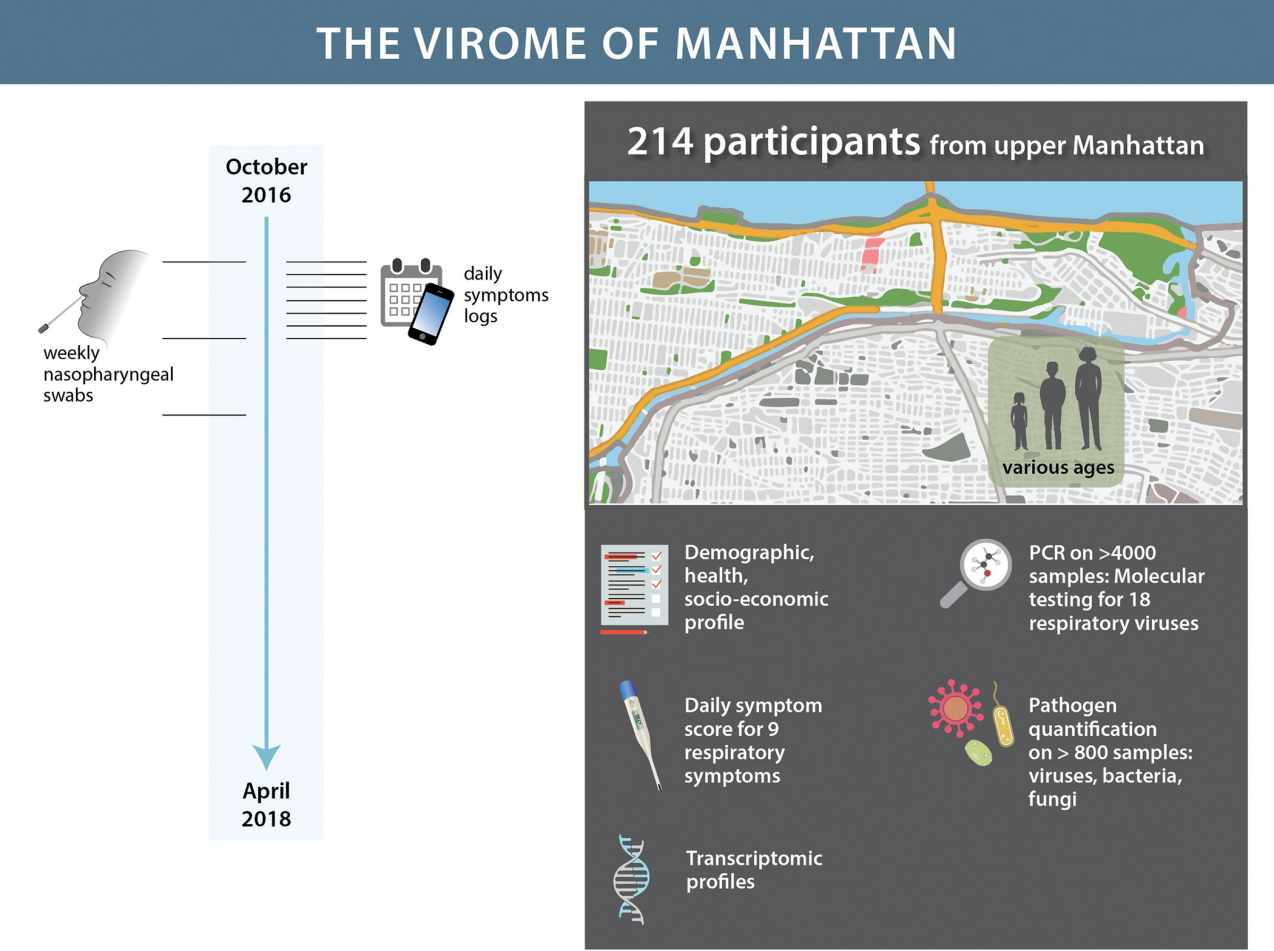
Description of The Virome of Manhattan Project. We enrolled a cohort of 214 participants from various locations in Manhattan for longitudinally studying the prevalence and characteristics of respiratory viral infections among the general (i.e., not hospitalized) population. At enrollment, we collected epidemiological information for each volunteer (demographic, health records, socioeconomic indicators). We collected weekly nasopharyngeal swabs over the course of 19 months. Using these specimens, we performed qPCR and RNA-seq data analyses for respiratory virus surveillance, transcriptomic comparisons, and detection of other pathogens. Simultaneously, we also tracked participant daily symptoms throughout the study. The Manhattan cartoon map in the figure is based on US Census Bureau map at: https://data.census.gov/map?q=New+York+city,+New+York&layer=VT_2021_160_00_PY_D1&loc=40.8394,-73.9136,z10.7284.

A cohort of 214 participants was enrolled from various locations in northern Manhattan between October 2016 and April 2018, as part of the *Virome of Manhattan Project*. The cohort, already described in [22–24], was used to conduct longitudinal sampling of viral respiratory infections among the general population and consisted of children from 2 daycares together with their siblings and one of their parents, teenagers and teachers from a high school, adults working at a medical center, and doctors from an Emergency Department (see **Fig 1**). At enrollment, all participants were younger than 65 years old; *n* = 35 (16%) were children under 10 years old, *n* = 42 (20%) were teenagers between 14 and 18 years old, and *n* = 137 (64%) were adults.

Two nasopharyngeal samples were collected once a week from all available participants by the study coordinators using minitip flocked swabs, irrespective of participant symptoms (see Methods section). Samples were then screened for viruses with a multiplex PCR assay for 18 commonly circulating respiratory viruses: rhinovirus, influenza A (H1N1, H1N1pdm2009, H3N2, and any subtype), influenza B, RSV (A and B) parainfluenza (1,2,3,4), coronavirus 229E, NL63, OC43, HKU1, adenovirus (B/E and C), and metapneumovirus. Daily reports of 9 respiratory symptoms (fever, chills, muscle pain, watery eyes, runny nose, sneezing, sore throat, cough, chest pain) self-evaluated on a Likert scale (0 = none, 1 = mild, 2 = moderate, 3 = severe) were recorded via a mobile app. The cumulative daily symptom score was defined as the sum of the 9 individual symptom scores (range: 0 to 27) on a given day. We used the 7 days surrounding the date of testing (Score7, i.e., the sum of individual symptoms scores from 3 days before to 3 days after the day of testing) to distinguish symptomatic from asymptomatic samples.

**Fig 2:**
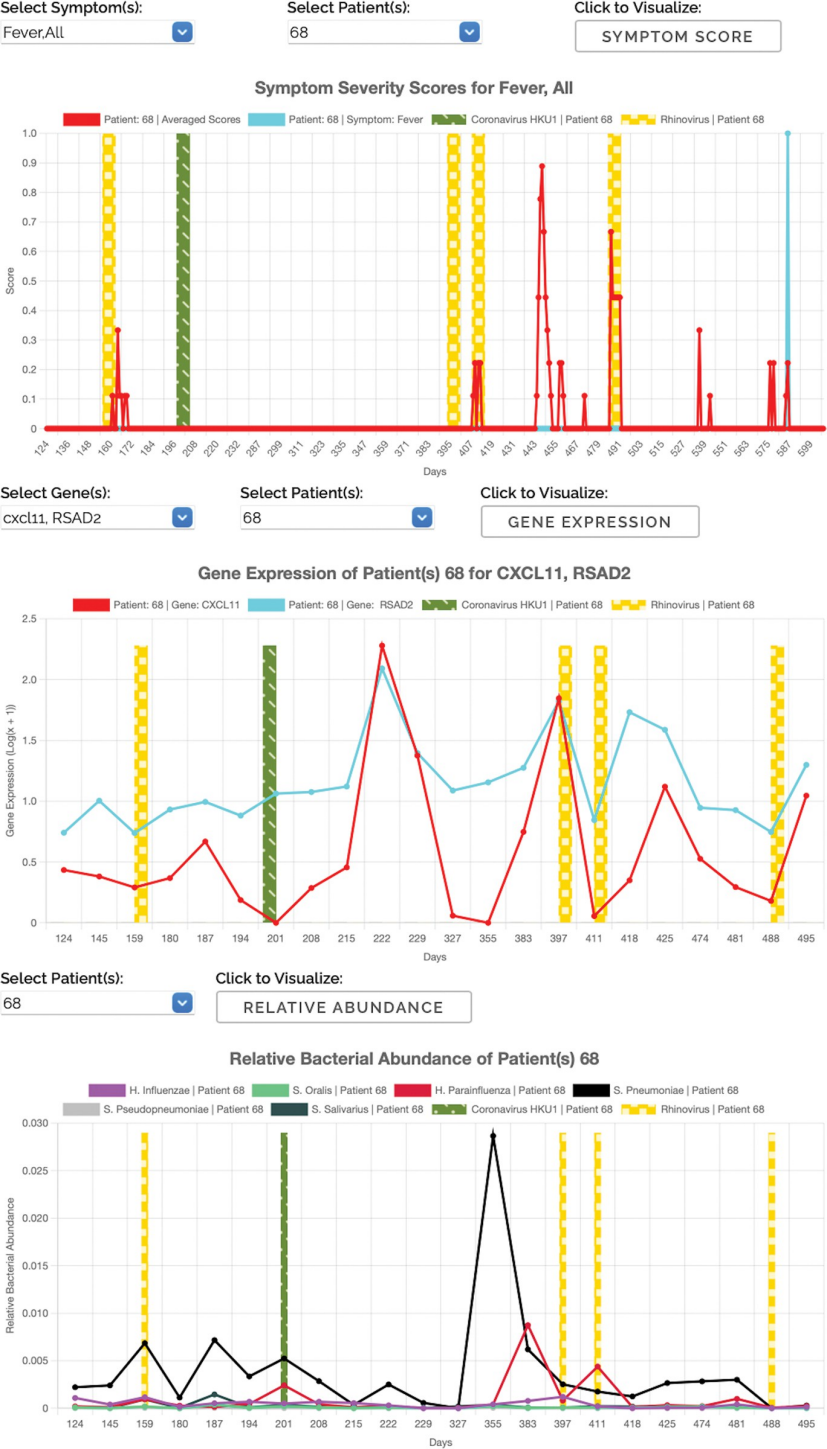
Example of longitudinal patient data extracted from the visualization website. *The Virome of Manhattan Project*: *Virome Data Explorer* **(screenshot):** for selected participant(s) a drop down menu visualizes self-reported individual and cumulative daily symptom score (first panel, the red line is here the daily longitudinal cumulative score normalized by number of symptoms), expression levels of a selection of genes measured in RNA-sequenced nasopharyngeal samples (second panel, the colored lines show here the expression level of 2 selected genes RSAD2, CXCL11), and the bacterial abundance of 6 selected bacteria (third panel, unit is bacterial reads relative to nonhuman-mapped reads in the RNA-seq sample). Vertical bars in all panels indicate viral infections detected via RNA-seq and time is measured in days from the beginning of study. Specific visualizations in Fig 2 correspond to selecting ID 68, symptoms: *fever*, *all* (i.e., cumulative) and genes *RSAD2*, *CXCL11* from the dropdown menu.

Among the 4,215 samples collected, we selected a subset of samples for RNA sequencing, based on several criteria including the presence of a respiratory virus as reported by the multiplex PCR assay and the quality of the sample (see Methods section). After eliminating duplicates and samples that failed sequencing runs, our dataset included 847 sequenced samples from 104 participants, with a median number of 7 samples per participant. RNA-Seq data was processed and analyzed to identify pathogens, host-specific transcriptional signatures, in silico immune cell decomposition, and bacterial coinfections (see Methods section). In **Fig 2**, we show an example of longitudinal data collected from an individual including weekly symptom score, expression level of a selection of immune-related genes, and the relative abundance of selected bacteria.

Of the 847 samples that were sequenced (see S1 Data for results and metadata), 237 were found positive for at least 1 respiratory virus, 597 were negative, and 13 were classified as undetermined and excluded from subsequent analysis (see Methods for positivity detection criteria). Among the 597 positive samples, 18 were coinfections of multiple respiratory viruses. **Table 1** lists the respiratory viruses that were isolated: the most frequent was rhinovirus (134), followed by coronaviruses (92), RSV (11), influenza viruses (8), WU Polyomavirus (6), parainfluenza viruses (2), adenovirus (2), human bocavirus (2), coxsackievirus (1), and enterovirus (1). Among the 237 samples testing positive for respiratory viruses, only 18 (8%) were positive for multiple respiratory viruses, with 5 viruses simultaneously isolated in one instance. Interestingly, all 6 occurrences of WU Polyomavirus were found in samples coinfected with other respiratory viruses.

**Table 1:** Viruses identified in the 847 sequenced samples, with associated family, genus, and type/group specification when available.

Family	Genus	Virus	Group/type	Total
*Picornaviridae*				
	*Enterovirus*			
		Enterovirus	undetermined	1
		Rhinovirus (HRV)		
			Rhinovirus A	42
			Rhinovirus B	39
			Rhinovirus C	53
		Human Coxsackievirus A		1
*Coronaviridae*				
	*Betacoronavirus*			
		Coronavirus OC43		42
		Coronavirus HKU1		16
	*Alphacoronavirus*			
		Coronavirus 229E		24
		Coronavirus NL63		9
*Pneumoviridae*				
	*Orthopneumovirus*			
		Respiratory syncytial virus (RSV)		
			RSV A	1
			RSV B	8
			undetermined	2
*Paramyxoviridae*				
		Parainfluenza (PIV)		
			PIV 2	1
			PIV 4	1
*Orthomyxoviridae*		Influenza		
			Influenza A	4
			Influenza B	2
			Influenza C	2
*Parvoviridae*				
	*Bocaparvovirus*			
		Human Bocavirus		1
*Adenoviridae*				
		Adenovirus	Adenovirus C	2
*Polyomaviridae*				
	*Betapolyomavirus*			
		WU Polyomavirus		6

### Dynamic transcriptional responses during natural infections

To understand how pathogen and host responses change through time in the course of natural infections, we selected 410 samples representative of longitudinal viral episodes (see Methods), among which 102 samples were from children (age <10), 52 were from teenagers (age 14 to 18), and 256 from adults; 157 samples were from male and 253 were from female participants. Samples were classified as PRE samples (*n* = 98, the last negative samples collected less than 21 days prior to an infection event), DURING samples (*n* = 166, the first sample of an infection event), DURING2 (*n* = 30, the second sample of an infection event), POST1 samples (*n* = 76, the first negative sample collected less than 11 days after the last positive), and POST2 (*n* = 42, the second negative sample collected 11 to 20 days after the last positive).

**Fig 3:**
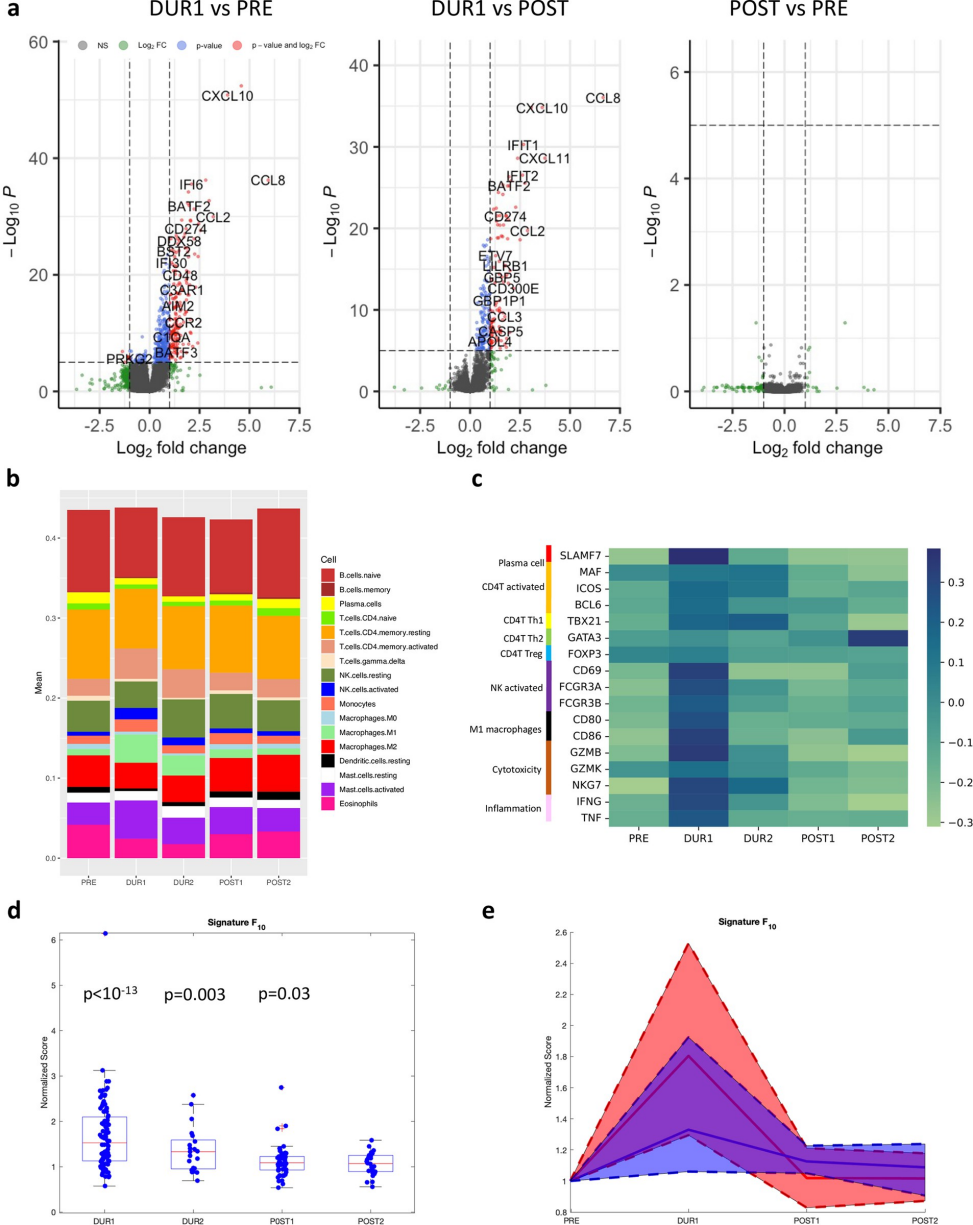
Dynamic transcriptional responses during natural infections. Panel (a): Volcano plots of differentially expressed genes between DURING1 and PRE, DURING1 and POST1, POST1 and PRE. The cutoff for fold change is >|2|; the default cutoff for *p-*value is 10^-6. (b) Relative abundance of immune cells, as predicted with Cibersort, across the PRE-DURING1-DURING2-POST1-POST2 samples. Significant pairwise comparisons are reported in S2 Data. (c) Heatmap of normalized expression of selected genes, known to be markers for immune cells, across PRE-DURING1-DURING2-POST1-POST2 samples. (d) Scores *F*_10_ of longitudinal samples, calculated using the top 10 genes differentially expressed in the DURING1 vs. PRE comparison, normalized by the respective PRE sample in each episode. Red boxplot lines are the median of distributions, and blue boxes are the interquartile. Whiskers extend to all non-outliers of the distribution. Outliers are represented with red + symbol. For each group, we reported *p*-values of a Wilcoxon signed rank test for the hypothesis that the dataset comes from a distribution with median = 1. (e) Medians (straight lines) and interquartile (shaded areas) of longitudinal symptomatic (red) and asymptomatic (blue) episodes normalized by the respective PRE sample. Underlying data for panels (b–e) can be found in S5 Data.

Our results show a strong immune signal during the acute phase of infection, followed by a rapid decay of transcriptomic signal after infection clearance: 6,594 genes were differentially expressed (padj <0.05) between the PRE and the DURING1 phase. Among the differentially expressed genes, those that were up-regulated during infection (*n* = 1,422) had a much stronger significance (**Fig 3A** and S2 Data) with 210 genes more than 2-fold up-regulated and 23 genes more than 4-fold up-regulated. Gene set enrichment analyses revealed that the most significant biological processes associated with viral infection were those related to adaptive immune response, cytokine production, acute inflammatory response, lymphocyte- and neutrophil-mediated immunity (Gene Set Enrichment Analysis (GSEA) adj, *p*-value <0.05; all enrichment scores >2.0; see S3 Data). The 10 most significantly overexpressed genes (7.5*10^−53^ <padj<1.8*10^−32^) were known markers of activation for immune response against viruses, such as CXCL9 and CCL8, CXCL10, CXCL11 and BATF2 (associated with proinflammatory M1 macrophages), RSAD2, GBP1 and IFIT3, IFI6 (interferon-regulated genes), SLAMF7 (marker for plasma cells); see S2 Data for all differentially expressed transcripts.

Conversely, the difference between the PRE and POST1 groups was minor with no genes differentially expressed after FDR correction (**Fig 3A** and S2 Data). Furthermore, we did not see evidence of overexpression of recovery genes in POST1 versus DURING1 samples and despite some residual effects in the postinfection samples (i.e., the slower recovery of genes related to CD4 Th1 activation (MAF, ICOS)), the profile of POST samples closely resembles that of PRE samples (**Fig 3C**). We did not find obvious functional annotation for down-regulated genes during infection, although many transcripts are consistent with previous reports [15].

These longitudinal data were further subjected to in silico cell sorting with CibersortX, in order to compare the relative abundance of different leukocytes. Our results suggest that onset of infection leads to an increase of proinflammatory macrophages M1 (Fold-Change = 4.4), activated CD4 T cells (FC = 1.8), and activated NK cells (FC = 2.9) (all Padj <0.05, Mann–Whitney tests) in nasopharyngeal samples. Soon after an infection is cleared, most changes are restored (S4 Data, **Fig 3B**, and corresponding S5 Data). We compared these findings with the changes in expression of gene markers for different immune cell types. In agreement with the results from Cibersort, samples during infection had higher levels of expression for genes such as Maf, Icos (CD4 T cell activation), Cd69, FCGR3 (NK cell activation), Cd80, CD86 (macrophages M), as well as markers of cytotoxicity (GZMB, GZMK) and inflammation (IFNG, TNF) (**Fig 3C** and corresponding S5 Data).

The signature scores *F*_*N*_, based on the top 10, top 25, and top 100 differentially expressed genes between DURING1 and PRE (see Methods) were able to distinguish infection and postinfection samples from the paired preinfection samples (see **Fig 3D** for *F*_10_ and S1 Fig for *F*_25_ and *F*_100_). To pair samples, we restricted to the (*n* = 98) longitudinal episodes that included a PRE sample, and we normalized the DURING and POST scores, simply dividing them by the score of their respective PRE. Results showed that in addition to being markedly distinguishable from DURING samples (median DUR1 normalized score = 1.53, *p* < 10^−13^ and median DUR2 normalized score = 1.33, *p* = 0.004), PRE samples were still weakly distinguishable from POST1 (median POST1 normalized score = 1.09, *p* = 0.03) but not from POST2 (median POST2 normalized score = 1.07, *p* = 0.12, significance assessed with Wilcoxon signed rank tests).

The effect of respiratory viral infections was not significantly different across sex and age group, even though some differences in the transcriptomic profile of immune genes across groups existed at baseline (i.e., in no-infection condition). Specifically, in the absence of viral infections, children displayed a larger expression of immune genes compared to adults that was significant for 94 of the 100 genes of signature F_100_. S7 Data and S2 Fig show the infection effect in male versus female and in children versus teenagers versus adults, together with pairwise comparisons between different groups at baseline.

We also compared the transcriptomic signal, quantified by *F*_10_ and *F*_100_, across normalized symptomatic and asymptomatic episodes (an episode was symptomatic if the weekly symptom score associated with any DURING sample was above 9 and asymptomatic otherwise, see Methods). The transcriptomic signal of the acute phase increased more markedly in symptomatic than in asymptomatic episodes (*p* = 0.01 and *p* = 0.003, Wilcoxon rank sum test); however, the recovery phase of POST1 and POST2 did not differ significantly across the 2 (**Figs 3E** and S3). In the next section, we investigate the association between symptom severity and differential expression of immune-related genes in greater depth.

In summary, our longitudinal analyses reveal that viral infection generally leads to a strong activation of immune response, followed by a rapid decay of the expression of immune-related genes once the viral infection has cleared.

### Specific transcriptional signatures are associated with symptom severity

To understand how asymptomatic infections differ from symptomatic ones, we compared the transcriptomic profiles of symptomatic and asymptomatic samples. We used a set of 699 samples (see sample selection in Methods), of which 209 were positive for at least 1 respiratory virus. This set of samples, featuring a median of 8 samples per individual, allowed comparison of the signal across symptom level and virus positivity status while controlling for the differences across the 81 contributing individuals. The samples were divided into 4 categories based on diagnosis (positive versus negative for respiratory viruses) and symptoms (symptomatic versus asymptomatic). Overall, there were 63 symptomatic positive (SP) samples, 146 asymptomatic positive (AP), 67 symptomatic negative (SN), and 423 asymptomatic negative (AN).

**Fig 4:**
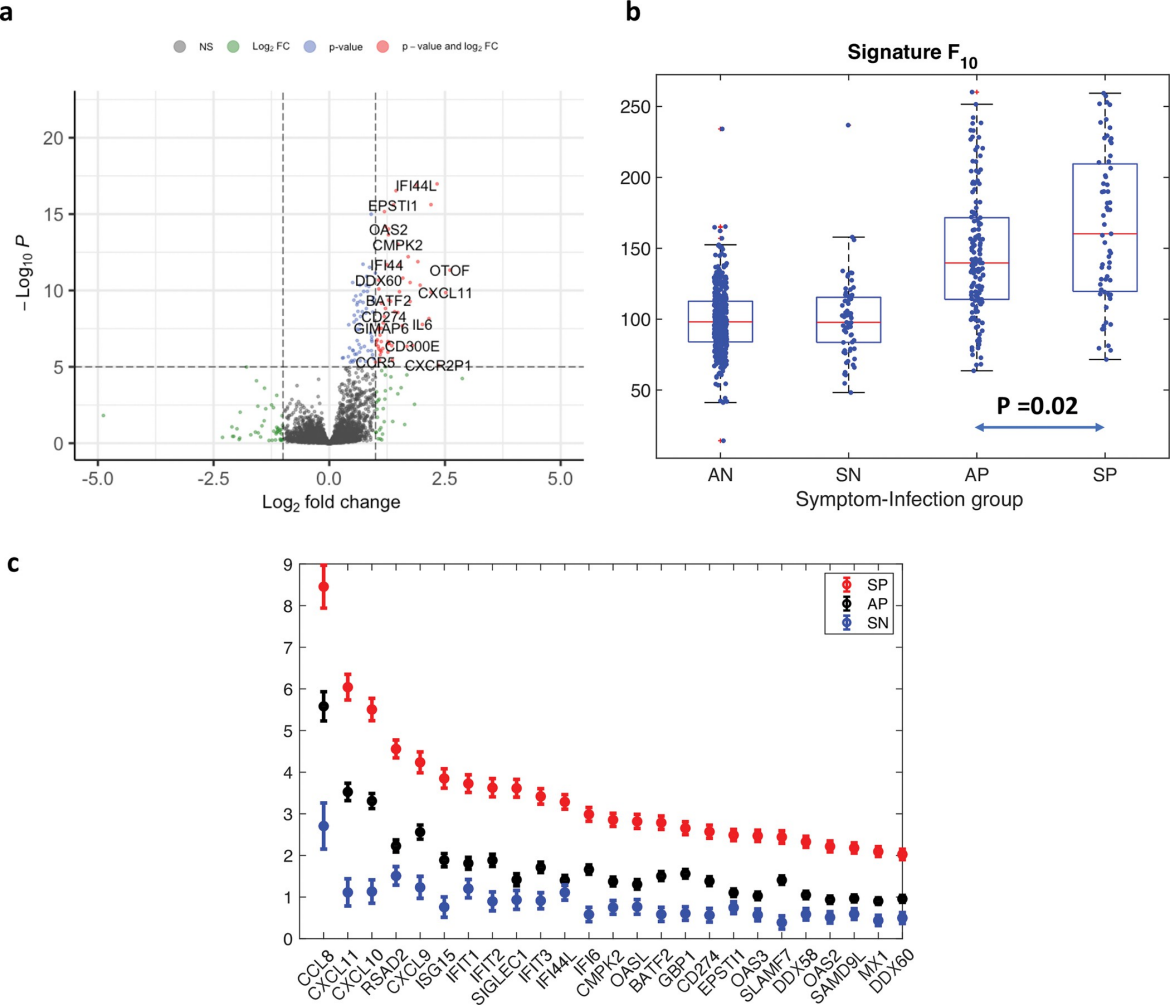
Specific transcriptional signatures are associated to symptom severity. **(a)** Volcano plots of differentially expressed genes between SP samples and AP samples. The cutoff for fold change is >|2|; the default cutoff for *P*-value is 10e-6**. (b)** Score factor F_10_ across the 699 samples calculated with the top 10 genes differentially expressed in the longitudinal analysis and applied to the 4 symptom-infection groups (AN, SN, AP, and SP). Samples expression values are normalized using the DESEQ2 normalization factor. Boxplots show the median (red line) and interquartiles (blue lines) of the distribution. Whiskers of boxplots extend to all non-outliers in the distribution. Outliers are shown as red “+”. **(c)** Log fold changes +/-SE for top DE genes in SP, AP, and SN when compared to AN samples assumed as baseline. The list of genes was selected as the top 25 DE genes in SP vs. AN. Panel (a) is based on data in S9 Data. Panels (b and c) are based on data in S10 Data. AN, asymptomatic negative; AP, asymptomatic positive; SN, symptomatic negative; SP, symptomatic positive.

We found 516 genes differentially expressed (419 overexpressed) in SP compared with AP (**Fig 4A** and S9 Data). The 81 genes that were at least 2-fold up-regulated at significance level *padj* <0.05 are well-known modulators of host immune response and included components of interferon pathways (MX1, IFITs, and IFIs groups), cytokines of the CXCL and CCL groups, genes with known antiviral activity (MX1, ISG15, RSAD2, and the OASs and DDXs groups), and genes associated with inflammation, such as IL6. These results were consistent when different thresholds were considered for the symptomatic classification: 506 (342 overexpressed) when using a symptom score threshold of >19 in SP versus AP (S11 Data). In this latter design, there were 43 SP samples, 166 AP, 26 SN, and 464 AN.

The top processes enriched in symptomatic positive versus asymptomatic positive, all with FDR < 0.05, included both innate and adaptive immune response processes (S12 Data), including immune response related to inflammation, such as the production of Type 1 Interferon, IL1, IL12, and IL6.

We then applied the expression signature score (factor score F_10_), calculated by combining the expression of the top 10 DE genes in the longitudinal analysis (CXCL11, CXCL10, CCL9, IFI6, IFIT3, CCL8, SLAMF7, BATF2, RSAD2, GBP1) to the 4 groups AN, SN, AP, and SP. The score F_10_ was significantly different among the groups of negative samples, positive asymptomatic, and positive symptomatic samples. In particular, the mean score for SP and AP was statistically different (Wilcoxon rank sum test, *p* = 0.02) and the mean score for AP samples was statistically different from both AN and SN (Wilcoxon rank sum test, *p* < 10^−32^ and *p* < 10^−13^, respectively). Results are consistent when using the metrics F_25_ and F_100_, respectively, calculated with the top 25 and top 100 genes (see S4 Fig).

We also statistically compared (with DESEQ2) the expression profiles of SP, AP, and SN to AN. The log2FC from a selection of the top 25 overexpressed genes in SP (from a total of 1,456 overexpressed genes; see S9 Data and S10 Data) shows a clear hierarchical structure in the 4 groups, with the log2FC of symptomatic positives on average 2 times that of the log2FC for asymptomatic positives (**Fig 4C**).

Overall, the severity of infection correlates with increased specific transcriptomic levels (see also analysis with symptom severity as a continuous variable in S11 Data). Interestingly, the expression profile of asymptomatic positives is markedly distinguishable from the negative samples (**Fig 4B and 4C**).

### Interactions between bacteria and respiratory viruses

We tested for potential viral and bacterial interactions during the course of natural infection. For this, we detected reads assigned to a number of bacterial species, common to the human respiratory tract, that can cause opportunistic diseases. We then assessed whether such bacterial abundance (measured as normalized fraction of reads) had a differential effect, at the host transcriptomic level, in individuals before and during a viral infection. This was done by performing differential gene expression analyses, using samples representative of longitudinal viral episodes (PRE and DUR1 samples).

**Fig 5:**
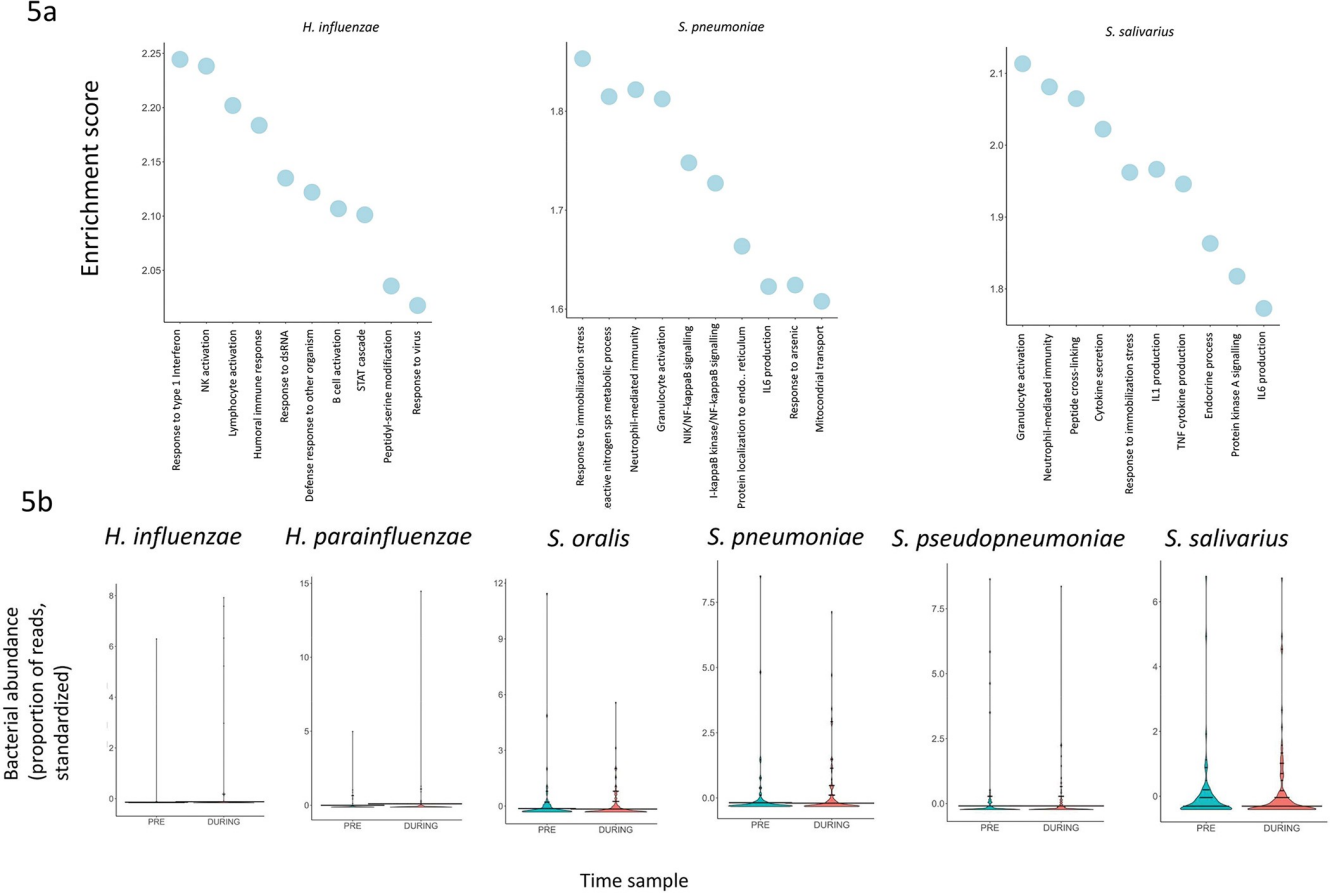
Interactions between bacteria and respiratory viruses. **Panel (a)**: Top 10 significantly enriched biological processes, as found with GSEA (FDR < 0.05), derived from the analysis of transcriptomic interactions between abundance of bacteria and viral infection. **Panel (b):** Comparison of bacteria abundance in PRE vs. DURING samples. Underlying data for Fig 5 can be found in S14 Data. GSEA, Gene Set Enrichment Analysis.

We found significant viral/bacterial interactions, at the gene expression level, for *Haemophilus influenzae* and 2 species of the genus Streptococcus: *S*. *pneumoniae* and *S*. *salivarus* (padjs <0.05 in DESEQ2 analyses). According to the GSEA results, the abundance of these bacteria in people infected with respiratory viruses was associated with higher expression of genes related to activation of lymphocytes (B cells, NK cells, and granulocytes) and interleukin (IL1, IL6) production (**Fig 5A** and S14 Data). Although we observed significant interactions at the transcriptomic level, abundance of these bacteria was not significantly different between positive and negative samples (**Fig 5B** and S14 Data).

In a similar way, we also assessed the interaction between symptomatology in viral infections and bacterial abundance. At gene expression level, we only found significant interactions between symptomatology and *S*. *pneumoniae*. According to GSEA, the abundance of these bacteria in symptomatic people infected with respiratory viruses was associated with higher expression of transcripts related to adaptive immune response (humoral immune response, B cell activate) as well as leukocyte proliferation and NK activation (S5 Fig and S15 Data). Within the participants infected with a respiratory virus, the abundance of bacteria was significantly different in symptomatic versus asymptomatic people only for *S*. *salivarius* and *S*. *oralis*, where there was a significant decrease of these bacteria in symptomatic samples (Mann–Whitney test: corrected *p*-value <0.05; S5 Fig).

### Identification of specific transcriptomic profiles of different viruses

**Fig 6:**
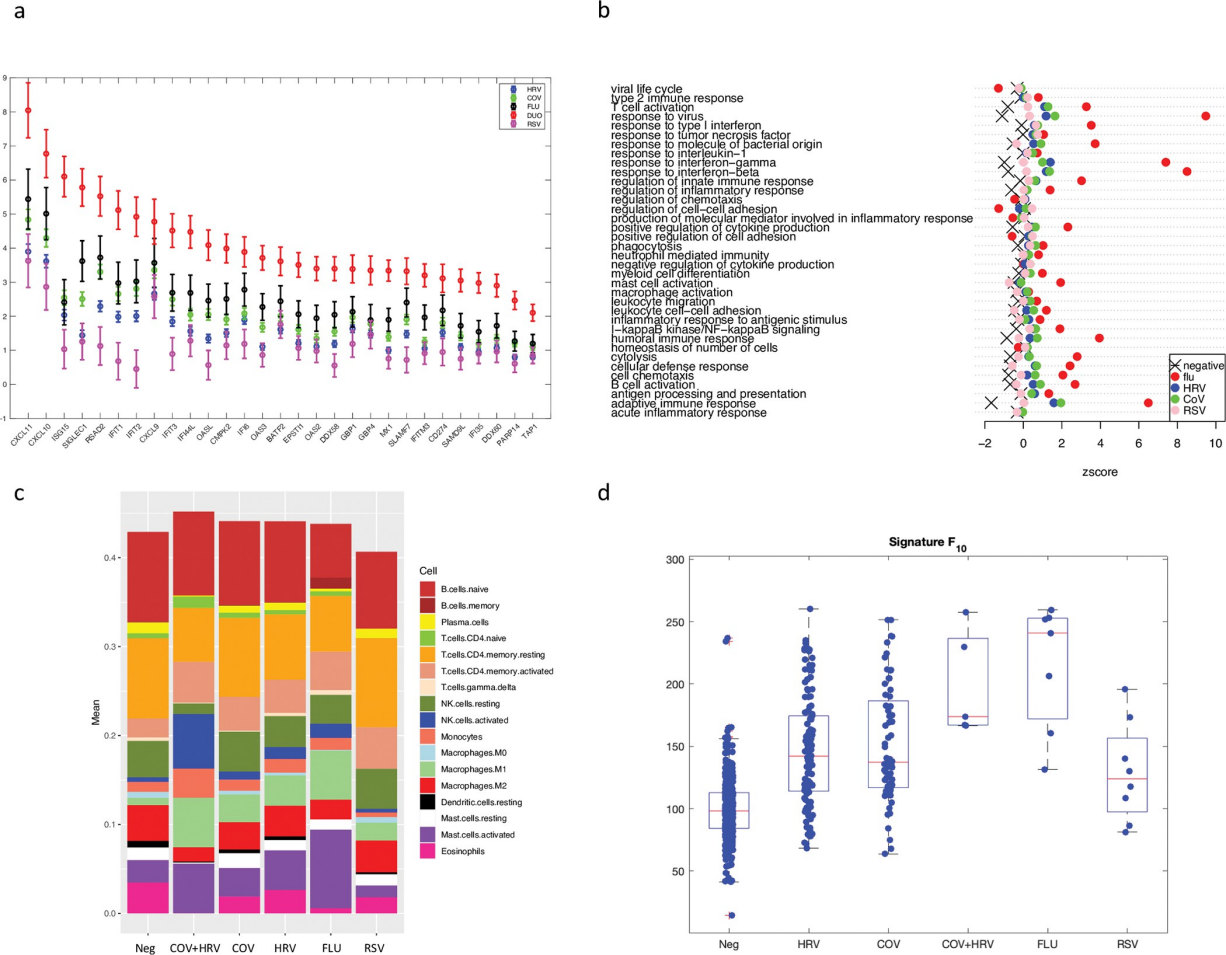
Identification of specific transcriptomic profiles of different viruses. (a) Log 2-fold change +/- SE of the expression of the top 30 DE genes across infection type (rhinovirus, coronavirus, coinfection rhinovirus/coronavirus, influenza, and RSV) with respect to negative samples. The top 30 genes were selected as the genes with lowest *padj* in the LRT comparing the full model (design~ *Batch+ParticipantID+Infection_type*) to the reduced model, in which the term *Infection_type* was removed. The significance was not corrected for FDR. (b) Comparison across different viruses of the top immune-related biological processes enriched during infection. Enrichment is quantified by the median zscore of samples that tested positive for each virus (colored dots) and compared to the median zscore across negative samples (black cross). Data underlying panel 6b are zscores reported in S19 Data. (c) Cibersort analysis of the relative abundance of cell types across infection type. Significant pairwise comparisons in cell abundance among viral infections are highlighted in S20 Data. (d) Score factor F_10_ across the 717 samples calculated with the top 10 genes differentially expressed in the longitudinal analysis and applied to the 6 viral infection groups: negative (Neg), HRV, CoV, CoV+HRV, influenza (FLU), and RSV. Boxplots show the median (red line) and interquartiles (blue lines) of the distribution. Whiskers of boxplots extend to all non-outliers in the distribution. Outliers are shown as red “+”. Panels (a), (c), and (d) are based on data in S17 Data. CoV, coronavirus; LRT, likelihood ratio test; RSV, respiratory syncytial virus.

To identify specific transcriptional signatures associated with specific viral infections, we compared the transcriptional profiles of the 5 most frequently isolated respiratory viral infections (rhinovirus, coronaviruses, coinfection rhinovirus/coronaviruses, influenza, and RSV) using a total of 717 samples. Other infection types were excluded from this analysis due to a low number of representative samples (i.e., less than 5 samples infected with a given virus). We found transcriptomic changes from baseline in previously identified immune genes for all infection types (**Fig 6A**). **Fig 6A** shows the log fold change in the 30 genes that showed the strongest change in expression across the infection type groups (see Methods). Pairwise comparison between baseline (*Neg)* and individual viruses are shown in S16 Data. Although many overexpressed genes were common to the different viruses, we observed differences across viruses in the magnitude of the transcriptomic response. Influenza was associated with a larger increase in the expression of immune-related genes. In contrast, RSV infections displayed the lowest change in expression from baseline (**Fig 6A**). Similar results were obtained when the analysis was repeated with only negative asymptomatic samples as baseline group (S18 Data). This observation is confirmed when comparing with Gene Set Variation Analysis (GSVA) the enrichment of immune-related processes across different viral infections (see Methods). The gene sets were selected as the immune processes that were overrepresented (*p* < 0.05) in the longitudinal and symptomatic analysis, and include activation of immune response, response to interferon and antiviral activity (**Fig 6B**).

Consistent with our previous results from longitudinal data, the CibersortX analyses stratified by virus revealed that viral infections lead to significant (Padj <0.05, Mann–Whitney tests) increases of activated CD4 T cells (Fold-Changes for CoV: 1.8—and HRV: 1.76), activated NK cells (FC for Flu: 2.06, HRV: 2.44, CoV+HRV: 12.44), and Macrophages M1 (significant for all viruses except RSV, FC ranging between 3.79 -CoV- and 6.79 CoV+HRV). We also found trends towards a decrease in plasma cells in all viruses except RSV (FC between 0.25 -Flu- and 0.68 -HRV-). Compared to HRV and CoV, influenza-infected samples had the highest relative abundance of activated Mast cells (mean relative abundances: flu = 0.088, HRV = 0.044, CoV = 0.032), M1 macrophages (mean relative abundances: flu = 0.055, HRV = 0.033, CoV = 0.031), NK activated cells (mean relative abundances: flu = 0.015, HRV = 0.013, CoV = 0.009), and activated CD4T cells (mean relative abundances: flu = 0.043, HRV = 0.037, CoV = 0.038) (**Fig 6C**, S17 and S20 Data files).

We then applied the expression signature score (factor score F_10_), calculated with the top 10 DE genes in the longitudinal analysis to the 5 groups of viral infection plus baseline. The score F_10_ significantly distinguished influenza samples from all other groups (**Fig 6D**) with the exception of coinfections CoV+HRV (Wilcoxon rank sum test, *p* = 0.02 with HRV, *p* = 0.03 with CoV, *p* = 0.04 with RSV). Results are consistent when using the metrics F_25_ and F_100_, respectively, calculated with the top 25 and top 100 genes (see S6 Fig).

There were no significant transcriptomic differences among infections with different rhinovirus species (A, B, and C), whereas among the 4 coronavirus types (OC43, HKU1, 229E, and NL63), CoV NL63 was associated with increased expression of immune-related transcripts. Moreover, several immune pathways were overrepresented in infections with alphacoronaviruses (C229E, NL63) compared to betacoronaviruses (OC43, HKU1), as shown in S7 Fig.

## Discussion

Viral respiratory infections are a major, global public health concern. Most people infected with seasonal respiratory viruses develop mild, self-resolving symptoms. However, in some cases, infections can lead to extremely serious complications, particularly in infants, elders, and immunocompromised hosts. The repeated emergences of influenza and coronavirus pandemic outbreaks (SARS, A/H1N1pdm2009, MERS, and SARS-CoV-2) have emphasized the need for a better understanding of host/pathogen interactions in respiratory infections. Classification of the main biological pathways affecting viral life cycle and host inflammation is key for developing therapeutic tools, such as antivirals or vaccines [25,26], as well as for better identifying individuals at risk, both for seasonally circulating respiratory viruses and emerging pandemic threats [27].

To investigate how a host responds to viral infection at the transcriptomic level, we designed a cohort study for the active surveillance of viral infections. Over the course of 19 months, we retrieved epidemiological, clinical (common respiratory-related symptoms), and molecular (RNA-seq and qPCR analyses from nasopharyngeal swabs) data over 100 volunteers enrolled within the Manhattan borough of NYC. As a result of this exhaustive work, we have built a rich dataset resource that provides a framework to study, longitudinally, the transcriptomic changes driven by different viral infections and across symptomatologies, as well as interactions between viruses and bacteria in the respiratory tract. Some previous transcriptomic analyses of respiratory infections have relied on symptomatic surveillance, selecting people with signs of respiratory illness [6,7,19–21], others have studied the transcriptomic changes driven by infection through in vitro analyses [9,12], and a number of designs have focused on challenge protocols using very small cohorts [13,14,16,17]. In our case, enrollment was not based on any specific risk factor, and weekly follow up of our large diverse cohort of child, teenage, and adult volunteers allowed detection of respiratory viral infection even in asymptomatic individuals. This is relevant because the vast majority of respiratory infections goes undetected, due to prevalent asymptomatic or mild disease [22] and individuals typically not seeking medical care for respiratory symptoms [23]. Nevertheless, there is a paucity of studies in the literature targeting asymptomatic and mild infection. We think that a combination of active surveillance programs, transcriptomic studies, such as the *Virome of Manhattan*, and designs such as [28], together with large-scale genome association studies and immunological studies will be critical for assessing the epidemiological impact and the biomarkers of asymptomatic infection in seasonal and pandemic outbreaks, and for helping identify protective mechanisms that can prevent severe outcomes of infections [29].

To facilitate access to the data derived from this study, we have made the results available through an interactive webserver searching for specific queries (e.g., transcript count distribution comparison between negatives and positives or between different symptomatic groups) and plotting longitudinal information of interest (e.g., how expression of genes of interest varies through time with symptomatology, highlighting time points when individuals tested positive for a given virus; see Fig 2). In total, we have collected information and analyzed data from 214 volunteers over the course of 19 months, resulting in over 800 sequenced samples. We designed this resource with the intention that users may generate and test new hypotheses.

As an example of the usefulness of the data resource, our longitudinal gene expression analyses have been able to replicate studies that previously revealed an increase in gene expression related to innate and adaptive immune response, T cells activation, and inflammation [15,17,20,30]. Among the most significantly up-regulated genes during respiratory viral infection, we found many chemokine genes (CXCL and CCL groups), interferon-induced genes (IFIT and IFI groups), and genes with known antiviral properties (RSAD2, MX1, ISG15, and OAS groups). Viperin (a protein encoded by RSAD2 gene) has been shown to reduce viral replication of HRV [15]. Deficiencies in MX1 and ISG15 and in genes of the OAS group have been associated with higher susceptibility to viral infections in mice [31,32]. There were also many down-regulated genes during infections; however, as in [15] we did not identify obvious functional annotations. We also showed that a simple factor score based on solely the top 10 overexpressed genes during infection found significant differences at the population level not only between infected and uninfected samples, but also between symptomatic and asymptomatic cases. Other signature factors have been used in the literature to characterize acute respiratory infections (ARIs) [16] and distinguish between viral and bacterial ARIs [4,17,18,30], and between symptomatic and asymptomatic infections [14,30].

Our dataset uniquely has longitudinal/paired data from viral episodes that also include samples within the 20 days before and 20 days after positivity. Most transcriptomic longitudinal analyses rely on experimental infections either in vivo or in vitro, and focus on the pre-symptomatic and acute phase of infection, limiting sampling to few hours/days postinfection [11,13,16,20]. In our cohort, samples infected with respiratory viruses presented a very strong response of immune genes with >1,400 genes overexpressed with respect to baseline preinfection samples. The steep decay of transcriptomic signal in postinfection samples was observed in both symptomatic and asymptomatic episodes and is in line with the rapid restoration shown in Zhai and colleagues at 21 days after symptom onset, when the transcriptomic profile of immunity genes in peripheral blood was not distinguishable from negative baseline, and with challenge studies that show a peak in gene expression 3 to 5 days postinoculation followed by a signal decrease [14,16,30]. We were not able to identify in the postinfection samples of our dataset the up-regulation of specific recovery genes found in [20], possibly due to a coarse sampling design (weekly, at most, but we allowed postinfections samples within 10 days from positive samples due to frequent sampling missed by participants). Zhai and colleagues employed a finer sampling design that enabled identification of a recovery phase between day 4 and day 6 following symptom onset.

The majority of the infections in our sample set were caused by rhinoviruses and coronaviruses. Only 8% of positive samples presented multiple viral coinfections, with one case positive for 5 different viruses. One of the viruses detected, WU Polyomavirus, was only found in coinfections with other respiratory viruses, confirming previously reported of high coinfection rates for this specific virus [33]. After comparing the transcriptomic changes driven by the most frequently isolated viruses (CoV, rhinovirus, influenza, RSV, dual infections with CoV and rhinovirus) and negative baselines, we observed a notable overlap of overexpressed genes. Many of the shared overexpressed genes have also been previously identified in SARS-CoV-2 infections [34] and across multiple seasonal respiratory viruses [8,17,30,35]. Rather than qualitative differences across viruses, we detected differences in the magnitude of differential expression of shared DE genes. Infection with influenza virus, for which the vaccination status of participants was unknown, was associated with stronger signal compared to infection with the other viruses. Specifically, CXCL11, RSAD2, IL6, IFIT1 presented more than 3 times the log fold change in influenza positive compared to rhinovirus positive samples. Other studies have reported a larger transcriptional response to influenza than RSV [9], rhinovirus (Dissanayake and colleagues), and multiple non-influenza respiratory viruses [20,28,30]. We also found increased expression of immune-related pathways in infections with alphacoronaviruses compared with infections with betacoronaviruses. This is particularly interesting as in the same cohort we found higher frequency of re-infections with betacoronavirus compared to alphacoronaviruses [36], suggesting a possible link between repeat exposure to pathogens and host responses. In our cohort, RSV infections presented the smallest up-regulation of immune genes, possibly due to the demographic of RSV infections (all the sequenced RSV–positive samples belonged to adults). Typically, people at risk for severe disease with RSV are infants and older adults, and some studies focusing on these demographics have found stronger effects [7].

Volunteers reported, on a daily basis, the presence and intensity of symptoms related to respiratory infections. These data, together with testing regardless of respiratory symptoms enabled us to characterize the transcriptomic signal of asymptomatic viral respiratory infections. Our results revealed not only that there are significant differences between symptomatic and asymptomatic individuals who are positive for respiratory viruses, but also that the transcriptomic signal of immune-related genes during asymptomatic/mild infection is strongly distinguishable from baseline (samples negative for respiratory viruses). This contrasts with previous studies that found no or little transcriptomic changes in asymptomatic or mild infections [13,17,28,30,37]. This discrepancy may partially depend on the lack of a uniform asymptomatic classification across studies. In our study, we only relied on self-reported symptoms and not signs (fever, for example, was reported as a symptom and did not require measurement) and parents reported symptoms for their small children. There was also very large variability of the average weekly symptom score across participants, with some individuals reporting no symptoms for the whole duration of the study and others with consistently very high scores [23]. To address this potential shortcoming, we ran several sensitivity analyses including different symptom score thresholds for classifying symptomatic and asymptomatic infection to ensure that our results were robust. The different model designs that we analyzed confirmed the presence of a strong transcriptomic response during asymptomatic and mild infections.

RNA-seq not only allows measurement and comparison of gene expression among samples, but also can be used to detect exogenous RNAs. In this work, we leveraged the RNA-seq data derived from our volunteers to assess positivity to respiratory viruses (in combination with qPCR results) and to measure the relative abundance of bacteria from the respiratory tract. In the online resource, we have included relative abundance of these bacterial species (bacterial reads relative to all reads not mapping to human, as obtained from the Pandora pipeline), as inferred by running Kracken/Bracken. We found significant transcriptomic interactions between respiratory viruses and different bacteria (*S*. *pneumoniae*, *S*. *salivarius*, and *H*. *influenzae*). Specifically, the abundance of these bacteria in samples positive for respiratory viruses was associated with higher expression of genes related to lymphocyte activation and granulocyte activity (e.g., phagocytosis, neutrophil activation) than in the absence of virus. We also found that the abundance of *S*. *pneumoniae* in samples from symptomatic individuals infected with respiratory viruses had higher expression of adaptive immune response and NK cell activation pathways than asymptomatic ones. Previous studies in mice and cellular cultures have searched for changes in immune response driven by coinfection by respiratory viruses (e.g., influenza) and bacteria such as *S*. *pneumoniae*. They have found a synergistic activation of proinflammatory cytokines that would lead to accumulation of immune cells such as neutrophils [38–42]. The findings that we obtained from our cohort are thus in line with these works and evidence interactions related to immune response between viral infections and the presence of common bacteria from the respiratory tract. While previous studies have mostly focused on the interaction between viral infections and *S*. *pneumonia*, we have demonstrated that similar interactions occur with other respiratory bacteria. Our public RNA-seq dataset will enable other investigators to identify RNA reads from other organisms of interest. One limitation of our study is that we did not account for medicine uptake during the course of the study. Specifically, antimicrobials use may have impacted the abundance of bacterial species and anti-inflammatory agents may have affected expression profile of target genes [43]. Vaccination status of the participants was also not accounted for.

In summary, we present a cohort study, consisting of hundreds of samples, that depicts the transcriptional changes driven by respiratory viral infection. In addition to the biological data derived from RNA-seq, for every volunteer we have collected information on other critical variables including age, sex, and daily symptoms. We have compiled these data to build a publicly available, user-friendly web-based resource where any user can compare, longitudinally over the course of 19 months, patterns of viral positivity, symptomatology and transcriptomic changes for the individuals enrolled.

## Methods

### Ethic statement

Study was approved by CUIMC IRB, protocol CUMC IRB AAAQ4358.

Participants of the study (or their guardians, if minors) provided written informed consent after reading a detailed description of the study.

### Specimen and symptoms data collection and classification

Duration of enrollment was heterogeneous for the participants: Some individuals remained enrolled for the entire study period (19 months), whereas others only for a few weeks (the shortest enrollment was 1 week). Demographic and enrollment information for all individuals in the *Virome Project* are summarized in [22,24] and S22 Data. Two nasopharyngeal samples were collected once a week from all available participants by the study coordinators using minitip flocked swabs, irrespective of the participants’ symptoms. Individuals were not always available for sampling collection, which resulted in incomplete series as described in [22,24]. The 2 samples were stored jointly in 2 mL DNA/RNA Shield (Zymo Research, Irvine, California, United States of America) at 4 to 25°C for up to 30 days and then stored at −80°C in 2 aliquots. Nucleic acids were extracted from 200 μl of sample and 10 μl of internal control using the EasyMAG NucliSENS system (bioMerieux, Durham, North Carolina, USA). Samples were then screened for viruses with the multiplex PCR assay *eSensor XT-8 respiratory viral panel* (RVP; GenMark Dx, Carlsbad, California, USA) [44]. The multiplex PCR assay tested for 18 commonly circulating respiratory viruses: rhinovirus, influenza A (H1N1, H1N1pdm2009, H3N2, and any subtype), influenza B, RSV (A and B) parainfluenza (1, 2, 3, 4), coronavirus 229E, NL63, OC43, HKU1, adenovirus (B/E and C), and metapneumovirus. Per manufacturer specification, samples PCR-positive for a particular virus were identified by an electrical signal intensity of ≥2 nA/mm^2^, except for coronavirus OC43, for which positive results were identified by an intensity of ≥25 nA/mm^2^. Demographic and enrollment information for all individuals in the *Virome Project* and features of the 4,215 samples collected are summarized in [22,24].

### Collection of RNA-seq data

Among the 4,215 samples collected, we selected a subset of samples for RNA sequencing. Selection criteria were: samples testing positive by the RVP for one of 18 respiratory pathogens along with corresponding preinfection and postinfection samples available for the same participant where at least 40% of the samples had RNA integrity score (RIN) above 4 as assessed with Agilent Bioanalyzer (Santa Clara, California, USA). For the selected samples, the remaining quantity of eluted RNA was sequenced with Illumina at the Columbia Genome Center with the Ribo-Zero rRNA Removal Kit, target 30 M single-end 100 bp reads for the first batch (Protocol *single* in model design) and 20 M paired-end 100 bp reads for the second batch (Protocol *paired* in model design).

Specifically, for single-end sequenced samples libraries were sequenced with Illumina HiSeq2500/HiSeq4000 at Columbia Genome Center. Samples were multiplexed in each lane, which yields targeted number of single-end/paired-end 100 bp reads for each sample, as a fraction of 280 to 400 million reads for the whole lane. RTA (Illumina) was used for base calling and bcl2fastq2 (version 2.17) for converting BCL to fastq format, coupled with adaptor trimming. The reads were mapped to human reference genome NCBI/build37.2 using STAR(2.5.2b) [45] and featureCounts (v1.5.0-p3) [46]. For paired-end sequenced samples libraries were sequenced with Illumina NovaSeq 6000 at Columbia Genome Center. Samples were multiplexed in each lane, which yields targeted number of paired-end 100 bp reads for each sample. RTA (Illumina) was used for base calling and bcl2fastq2 (version 2.20) for converting BCL to fastq format, coupled with adaptor trimming. A pseudoalignment was performed to a kallisto index created from transcriptomes (Human: GRCh38) using kallisto (0.44.0) [47].

After eliminating failed and duplicate samples, there were 847 unique samples sequenced from 104 different individuals, with a median number of 7 samples per participant (see S1 Data for details on number of samples per individual and date of collection).

### Quality control and pathogen detection

Quality control of the raw reads was assessed with FASTQC (see sequence quality plots in S9 Fig; underlying data for this figure available at GEO accession number GSE223679) [48]. In order to differentiate between human and nonhuman reads, and quantify reads related to respiratory viruses, we ran the pipeline Pandora: an open-source pipeline developed by the Rabadan lab that has been validated and used previously [49]. The Pandora workflow takes RNA-seq data as input and produces the annotated microbial reads present in the sample as outputs. The pipeline runs through 5 main modules: (1) mapping to the human host genome using STAR [45] and bowtie2 [50]; (2) processing of the host-mapped reads with featureCounts [46] to obtain the host gene expression profile; (3) de novo assembly of host-subtracted short (2 × 100 bp) reads using Trinity [51] to create long contiguous full-length transcripts (contigs); (4) contigs alignment to curated sets of NCBI sequences from viruses, bacteria, fungi, and other eukaryotic parasites with BLAST [52]; and (5) filtering and parsing the BLAST results into a report specifying the detected microbial abundances in the sample.

To assess positivity for respiratory viruses in the 847 sequenced samples we first isolated pathogen reads attributed to human viruses falling in the picornaviruses, adenoviruses, polyomaviruses, parvoviruses, coronaviruses, paramoxyviruses, and pneumoviruses families. We then set a reads threshold for assessing positivity to correct for (1) short reads fragments typically present in metagenomics samples; and (2) index hopping, a phenomenon occurring in multiplex sequencing at different scales for single and paired-end protocols (typically 2 orders of magnitude different [53]). The threshold was set different for samples testing positive and negative by qPCR (qPCR+ and qPCR-). Samples qPCR+ for a specific family of viruses were considered positive if containing at least 15 reads of a virus in that family and negative otherwise. Samples qPCR- were considered negative for a respiratory virus if containing less than 500 reads of that respiratory virus. Conversely, qPCR- samples with more than 500 reads of a virus were considered positive for that virus, with an additional correction for index hopping that became relevant from samples with over 10^5 reads of a specific virus. More specifically, the threshold of positivity for a virus was max(500, IH*10^-3) for single-end protocols and max(500, IH*10^-5) for paired-end protocols, with IH being the largest reads count of that virus within samples the same batch. Samples qPCR- containing more than 500 reads but less than max(500, IH*10^-3) for single-end or max(500, IH*10^-5) for paired-end were considered *Undetermined* and excluded from subsequent analysis.

In S8 Fig, we evaluate the agreement of qPCR and RNAseq for different RNAseq thresholds using only viruses included in the RVP multiplex.

We defined an infection event as a group of consecutive weekly specimens from a given individual that were positive for the same virus. Samples from the same individual that were positive for the same virus were considered part of the same infection event if separated by a week gap (i.e., a missing test). Coinfections (samples positive for multiple respiratory viruses) were considered one infection event if all samples of the event were positive for the same viruses, or if later samples were positive for a subset of viruses (e.g., *coronavirus+rhinovirus* on week 1 and *rhinovirus* only on week 2). Conversely, if a new virus was detected in addition to a previous one (e.g., *rhinovirus* on week 1 and *coronavirus+rhinovirus* on week 2), we considered the infections as separate events.

Nonhuman reads were further used to identify and quantify bacterial species commonly found in the respiratory tract (*H*. *influenzae*, *H*. *Parainfluenziae*, *S*. *pneumoniae*, *S*. *Pseudopneumoniae*, *S*. *Oralis*, *S*. *Salivarius*). These reads were analyzed using Kracken [54], a taxonomic sequence classifier that analyzes short reads, in combination with Bracken, a companion program to Kracken that accurately classifies and quantifies reads assigned at species resolution using a Bayesian approach [55].

**Fig 1** describes the structure of the complete *Virome* dataset leveraged in this analysis and available at the web-resource *The Virome of Manhattan Project*: *Virome Data Explorer*.

### Gene expression analyses

We used the R package DESEQ2 to perform group comparison of differentially expressed genes, using the Wald test for hypothesis testing in the principal analysis, and the likelihood ratio test (LRT) to evaluate design models [56]. Pairwise differences between groups were quantified with the log2fold change of the expression levels of 18,000+ genes, evaluated using *p*-values corrected for multiple testing using the methods of Benjamini and Hochberg (*padj*). We set the threshold for significance of the adjusted *p*-values at padj <0.05.

We performed the following comparisons:

- **Longitudinal analyses on samples collected before, during, and after an infection event**.

We defined longitudinal episodes by selecting infection events of individuals that presented pre- and postinfection samples, as described hereafter. In a longitudinal episode, DURING1 was the first positive sample in an infection event, DURING2 was a subsequent positive sample belonging to the same infection event, PRE was a negative sample collected less than 21 days before the first positive sample of an event and with no known positivity in the previous 2 weeks, POST1 was a negative samples collected less than 11 days after the last positive test of an event and POST2 was a negative sample collected between 11 and 20 days after the last positive test of an event and following a POST1 sample. We allowed for incomplete longitudinal sets of samples, but only participants that contributed at least 1 positive (DURING1) and 1 corresponding negative sample (either a PRE or a POST1) were included in this analysis. The selected set consisted of 410 samples, including 98 PRE, 166 DURING1, 30 DURING2, 76 POST1 42 POST2.

We used a linear model that considered the variables “Patient ID”, “Time_Sample {Categories: PRE/DURING1/DURING2/POST1/POST2}” and “Batch {paired-end sequency technology/single-read sequencing technology}” to analyze pairwise comparisons among the *Time_Sample* category. We further analyzed the differential impact of infection across sex (categories male/female) and age groups (categories children/teenager/adults) using DESEQ designs with interaction terms.

We also compared the 5 group categories by creating a gene expression signature score, based on the combination (summation) of the top differentially expressed genes identified in the DURING1 versus PRE comparison. This signature score, which enables profiling of each sample, was calculated for each sample as $F_{N}(i)=\sum_{g=1}^{N}w_{g}log(x_{g}(i)+1)$, where the weight *w*_*g*_ is the Log2 Fold Change of gene *g*, as estimated in DURING1 versus PRE, *x*_*g*_(*i*) is the expression of gene *g* in sample *i*, normalized by size factors (sample-specific size factors in DESEQ2 determined by median ratio of gene counts relative to geometric mean per gene). Multiple cutoffs values were considered for N (*n* = 10, *n* = 25, *n* = 100) based on the *padj* ranking. We used Wilcoxon signed rank test to compare the distribution of factor scores across paired groups, and Wilcoxon rank sum test for comparisons across unpaired groups.

- **Differences related to symptomatology**. Symptomatic and asymptomatic individuals were compared, differentiating among positivity status (samples testing positive or negative for respiratory viruses). We defined a sample symptomatic if the associated cumulative symptom score was >9 and asymptomatic otherwise. To compare transcriptomic profiles across positivity and symptom status while controlling for individual variability, we used all samples (*n* = 699) that (1) had associated symptom scores; and (2) belonged to participants that contributed at least 1 infection event and that also presented at least 1 negative sample (not necessarily in the same longitudinal episode). The samples were classified into 4 categories of “sample type”: asymptomatic negative (AN), symptomatic negative (SN), asymptomatic positive (AP), symptomatic positive (SP). We used a linear model with the variables “Batch”, “Patient ID”, and “Sample_Type”. Two additional designs, one that considered a sample symptomatic if the associated cumulative score was >19 and one that considered the symptoms as a continuous variable (log-transformed), were also analyzed and are presented in S11 Data. A sensitivity analysis, performed by eliminating the influenza positive samples, is also shown in S11 Data.

We also compared the 4 groups (AN, SN, AP, and SP) using the same gene expression signature scores *F*_*N*_(*i*) from the longitudinal analyses and the same weights based on the top 10, 25, and 100 differentially expressed genes in the pairwise DURING1 vs. PRE analysis with DESEQ2.

- **Interaction between virus infection and pathogenic bacteria**. We tested, for a set of bacteria commonly found in the respiratory tract (*H*. *influenzae*, *H*. *Parainfluenziae*, *S*. *pneumoniae*, *S*. *Pseudopneumoniae*, *S*. *Oralis*, *S*. *Salivarius*), whether their presence was associated with differential effect, at the transcriptomic level, in individuals before and during a viral infection. For this analysis, we used a model with the variables “normalized bacterial reads”, “Patient ID”, “batch” and “Time_Sample {PRE/DURING1}”, that included an interaction term between “normalized bacterial reads” and “Time_Sample”. Note that all variables are categorical, with exception of “normalized bacterial reads”, which is a standardized continuous variable. In addition, we investigated the interaction between bacterial abundance and symptomatology. For this, we used a similar model with an interaction term between the variable “normalized bacterial reads” and the categorical combinations (AN, SN, AP, and SP) described in previous paragraph that considered the joint occurrence of viral infection and symptomatology.
- **Gene expression changes related to each virus type.** We compared the transcriptional profile of the 5 most frequent infection types (CoVs, Rhinovirus, Flu, RSV, or dual infection with rhinovirus and CoVs). For this, we selected all available samples (*n* = 717) from individuals contributing *at least* 1 negative (baseline) sample and 1 infection event among these 5 infection types. Using the samples negative for all viruses as baseline, we searched for genes that differentially express in response to the different viruses (CoVs, Rhinovirus, Influenza, RSV, dual infection). The model considered a linear combination of the variables “Patient ID”, “Batch” and “infection_type” {negative, COV, HRV, Influenza, RSV, COV+HRV}. For this analysis, we excluded coinfections (samples testing positive for multiple respiratory viruses). We further analyzed the differences within rhinovirus species (A, B, C) and Coronavirus types (C229E, NL63, OC43, HKU1) and genera (alphacoronaviruses, including C229E and NL63, and betacoronaviruses, including OC43 and HKU1). We identified the 30 genes displaying the largest transcriptomic changes across infection type by comparing the full model (design~ *Batch+ParticipantID+Infection_type*) to the reduced model, in which the term *Infection_type* was removed, with the LRT of DESEQ2.

We used over-representation analysis (ORA), GSEA from WebGestalt (WEB-based Gene SeT AnaLysis Toolkit, [57]), and GSVA [58], from the *GSVA* R package, to interpret the enriched gene list, identify relevant biological processes affected by the differential expression, and compare them across conditions. When using GSEA, we ranked all genes of the count matrices according to the metric (-log(pvalue))*sign(log2FoldChange), where log2FoldChange is the log fold change of expression value with respect to baseline. *P*-value was calculated with the Wald test. With GSVA, we mapped the variance stabilized transformation (VST) of the count table into a gene set by sample matrix using method *zscore* [59] and we used the *zscore* metric, which combines across predefined gene sets the standardized gene expression profiles, to compare the relative enrichment of immune-related pathways across different viruses.

All RNA-seq samples were normalized to reads per kilobase per million (RPKM) before performing an in silico cell sorting with CibersortX [60] to estimate the relative abundance of immune cell types. This deconvolution was done using a validated leukocyte gene signature matrix of human origin, LM22. This matrix, which contains 22 functionally defined human hematopoietic cell types, was generated using Affymetrix HGU133A microarray data [61]. Wilcoxon rank tests were performed to assess the significance of different abundances of specific cell types between negative and infected samples and between different viral species.

## Supporting information

S1 Fig Factor scores longitudinal. Distribution of scores for **(a)** Factor_10_, **(b)** Factor_25_, **(c)** Factor_100_ calculated respectively with the top 10, 25, and 100 genes differentially expressed in the longitudinal analysis of DURING1 vs. PRE. Median (red lines) and interquartile (blue boxes) of factor scores distributions are represented for the 5 longitudinal points PRE, DURING1, DURING2, POST1, and POST2. Figure is based on data in S6 Data.
(TIF)

S2 Fig Age and sex effect. Comparison across age (children vs. adults) and sex groups (male vs. female) at baseline (i.e., in samples negative for respiratory viruses) and in positive samples (infection effect over baseline). Volcano plots were restricted to the top 100 immune genes overexpressed in the longitudinal analysis, used to build the metric F_100._ Figure is based on data in S7 Data.
(TIF)

S3 Fig Results factor score *F*_100_. **(a)** Medians (straight lines) and interquartile (shaded areas) of scores based on *F*_100_ for longitudinal symptomatic (red) and asymptomatic (blue) episodes normalized by the respective PRE sample. **(b)** Scores *F*_100_ of longitudinal samples, calculated using the top 10 genes differentially expressed in the DURING1 vs. PRE comparison, normalized by the respective PRE sample in each episode. Red lines in boxplot are the median of distributions and blue boxes are the interquartile. Whiskers extend to all non-outliers of the distribution. Outliers are represented with red + symbol. For each group, we reported significant *p*-values of a Wilcoxon signed rank test for the hypothesis that the dataset comes from a distribution with median = 1 at the 5% significance level. Figure is based on data in S8 Data.
(TIF)

S4 Fig Factor scores symptoms. Distribution of scores for Factor_10_, Factor_25_, Factor_100_ calculated respectively with the top 10, 25, and 100 genes differentially expressed in the longitudinal analysis of DURING1 vs. PRE. Medians (red lines) and interquartile (blue boxes) of factor scores distributions are represented for the 4 groups of asymptomatic negative (AN), symptomatic negative (SN), asymptomatic positive (SP), and symptomatic positive (SP). Figure is based on data in S13 Data.
(TIF)

S5 Fig Interactions between bacteria and symptomatology in samples positive for respiratory viruses. **Panel (a)**: Top 10 significantly enriched biological processes, as found with GSEA (FDR < 0.05), derived from the analysis of transcriptomic interactions between abundance of bacteria and viral symptomatic viral infection for *S*. *pneumoniae* (only bacteria with significant association). **Panel (b):** Comparison of bacteria abundance in symptomatic vs. asymptomatic samples. Figure is based on data in S15 Data.
(TIF)

S6 Fig Factor scores individual viruses. Distribution of scores for Factor_10_, Factor_25_, Factor_100_ calculated respectively with the top 10, 25, and 100 genes DE in the longitudinal analysis of DURING1 vs. PRE. Medians (red lines) and interquartile (blue boxes) of factor scores distributions are represented for plus negative baseline samples (None) and for the 5 viral infection types analyzed (rhinovirus (HRV), coronavirus (COV), coinfection of coronavirus and rhinovirus (COV+HRV), influenza (FLU), and respiratory syncytial virus (RSV)). Figure is based on data in S21 Data.
(TIF)

S7 Fig Comparison between alphacoronaviruses and betacoronaviruses. Comparison across the top immune-related biological processes enriched during infection (GSVA). Enrichment is quantified by the median zscore of samples that tested positive for each virus (colored dots) and compared to the median zscore across negative samples (black cross). Preselected processes are those overrepresented in the longitudinal and symptomatic analyses, as in Fig 6B. Figure is obtained applying GSVA to count data normalized with DESEQ (code and count data table in repository at *https*:*//github*.*com/RabadanLab/ViromeofManhattan*).
(TIF)

S8 FigComparison between qPCR and RNAseq.Only viruses included in the RVP are included in this comparison. First panel shows the percent of samples testing positive with qPCR, per increasing thresholds of RNAseq positivity (minimum number of reads of the virus identified by qPCR found in the sample). In formula, that is $\frac{{qPCR}^{+}(RNAseq>x)}{RNAseq>x}$. The second panel quantifies the percentage of PCR positivity *below* different threshold of RNAseq positivity. In formula, that is $\frac{{qPCR}^{+}(RNAseq<x)}{{qPCR}^{+}}$. The vertical black line indicates the threshold chosen in our analysis. Figure is based on data in S23 Data.(TIF)

S9 Fig FASTQC. FASTQC summary plot of the reads quality for samples processed with *single-end* protocol and *paired-end*. Figure obtained by running FASTQC on raw files submitted on GEO repository with accession number GSE223679.
(TIF)

S1 Data Samples_Metadata. List and associated metadata for the 847 sequenced samples in The Manhattan Virome Project. Metadata include self-reported age, sex, race, ethnicity, cohort of the participants and infection type, date of collection, and RNA protocol used for each sample. In the Sample_Id field, P identifies the participants and S identifies their samples in chronological order.
(CSV)

S2 Data Results_longitudinal. DESEQ2 results tables for the longitudinal analysis. Results for comparison between DURING1 vs. PRE (**resD1vsPR_longitudinal**), POST1 vs. PRE (**resP1vsPR_longitudinal**), and DURING1 vs. POST1(**resD1vsPOST1_longitudinal**).
(XLSX)

S3 Data GSEA_longitudinal. Enriched processes with padj <0.05 for D1vsPRE.
(XLSX)

S4 Data Cibersort_longitudinal. Statistics on pairwise comparisons in longitudinal analysis.
(XLSX)

S5 Data Data underlying Fig 3B–3E. (XLSX)

S6 Data Data underlying S1 Fig. (XLTX)

S7 Data Age and Sex effect. DESEQ2 results tables for the comparison across age groups (child_vs_adult, teen_vs_adult, child_vs_teen) and sex (male _vs_female) in baseline (no-infection) condition and for the infection effect (interaction term in DESEQ2).
(XLSX)

S8 Data Data underlying S3 Fig. (XLSX)

S9 Data Results_symptomatic. DESEQ2 results tables for the symptomatic analysis. Here, the threshold for symptomatic classification is Score7>9. Results for comparison between asymptomatic positive vs. asymptomatic negative (**APvsAN**), symptomatic negative vs. asymptomatic negative (**SNvsAN**), symptomatic positive vs. asymptomatic negative (**SPvsAN**) and symptomatic positive vs. asymptomatic positive (**SPvsAP**).
(XLTX)

S10 Data Data underlying Fig 4B and 4C. (XLTX)

S11 Data Results_ alternative_symptomatic. DESEQ2 results tables for the symptomatic analysis in alternative designs (1) **Score19**: symptomatic threshold is Score7>19, (2) **Score7Cont:** cumulative symptom score Score7 is a continuous metric, (3) **NoFLU**: flu samples are excluded from the analysis.
(XLSX)

S12 Data GSEA_symptomatic enriched processes with padj <0.05 for SP vs. AP. (XLSX)

S13 Data Data underlying S4 Fig. (XLTX)

S14 Data Data underlying Fig 5. (XLTX)

S15 Data Data underlying S5 Fig. (XLTX)

S16 Data Results_individual_Viruses. DESEQ2 results tables for the transcriptomic analysis by virus. Results for comparison between rhinovirus vs. negative for all respiratory viruses (**HRV_vs_none**), coronavirus vs. negative for all respiratory viruses (**COV_vs_none**), coinfections between rhinovirus and coronavirus vs. negative for all respiratory viruses (**HRVandCOV_vs_none**) and influenza vs. negative for all respiratory viruses (**FLU_vs_none**).
(XLSX)

S17 Data Data underlying Fig 6A, 6D and 6D. (XLTX)

S18 Data Results_ alternative_individual_viruses. DESEQ2 results tables for the individual viruses analysis in alternative designs (1) **LRT** between model with and without specification of type_infection (here type_infection does include negative samples), (2) **Virus_vs_asymptomatic_negative:** baseline comparison is performed using only asymptomatic negative sample.
(XLSX)

S19 Data Data underlying Fig 6B. (XLTX)

S20 Data Cibersort individual_viruses. Cibersort statistics on individual viruses.
(CSV)

S21 Data Data underlying S6 Fig. (XLSX)

S22 Data Cohort. Age and sex of participants included in this study.
(DOCX)

S23 Data Data underlying S8 Fig. (XLTX)

S24 Data Symptoms_scores (zip file). Daily score for 9 symptoms for the 104 participants, longitudinal records, organized by cohort (MC = medical center, D1 = daycare1, D2 = daycare2, SC = high school, ER = ER doctors).
(ZIP)

## Abbreviations

AN: asymptomatic negative
AP: asymptomatic positive
ARI: acute respiratory infection
CoV: coronavirus
GSEA: Gene Set Enrichment Analysis
GSVA: Gene Set Variation Analysis
LRT: likelihood ratio test
ORA: Over-representation analysis
RIN: RNA integrity score
RPKM: reads per kilobase per million
RSV: respiratory syncytial virus
SARS-CoV-2: Severe Acute Respiratory Syndrome Coronavirus 2
SN: symptomatic negative
SP: symptomatic positive
VST: variance stabilized transformation
