# Supplementary figures and images for: Virome Data Explorer: A web resource to longitudinally explore respiratory viral infections, their interactions with other pathogens and host transcriptomic changes in over 100 people
Source: PLoS Biol. 2024 Jan 18;22(1):e3002089. doi: 10.1371/journal.pbio.3002089 (PMC10796020; doi:10.1371/journal.pbio.3002089)

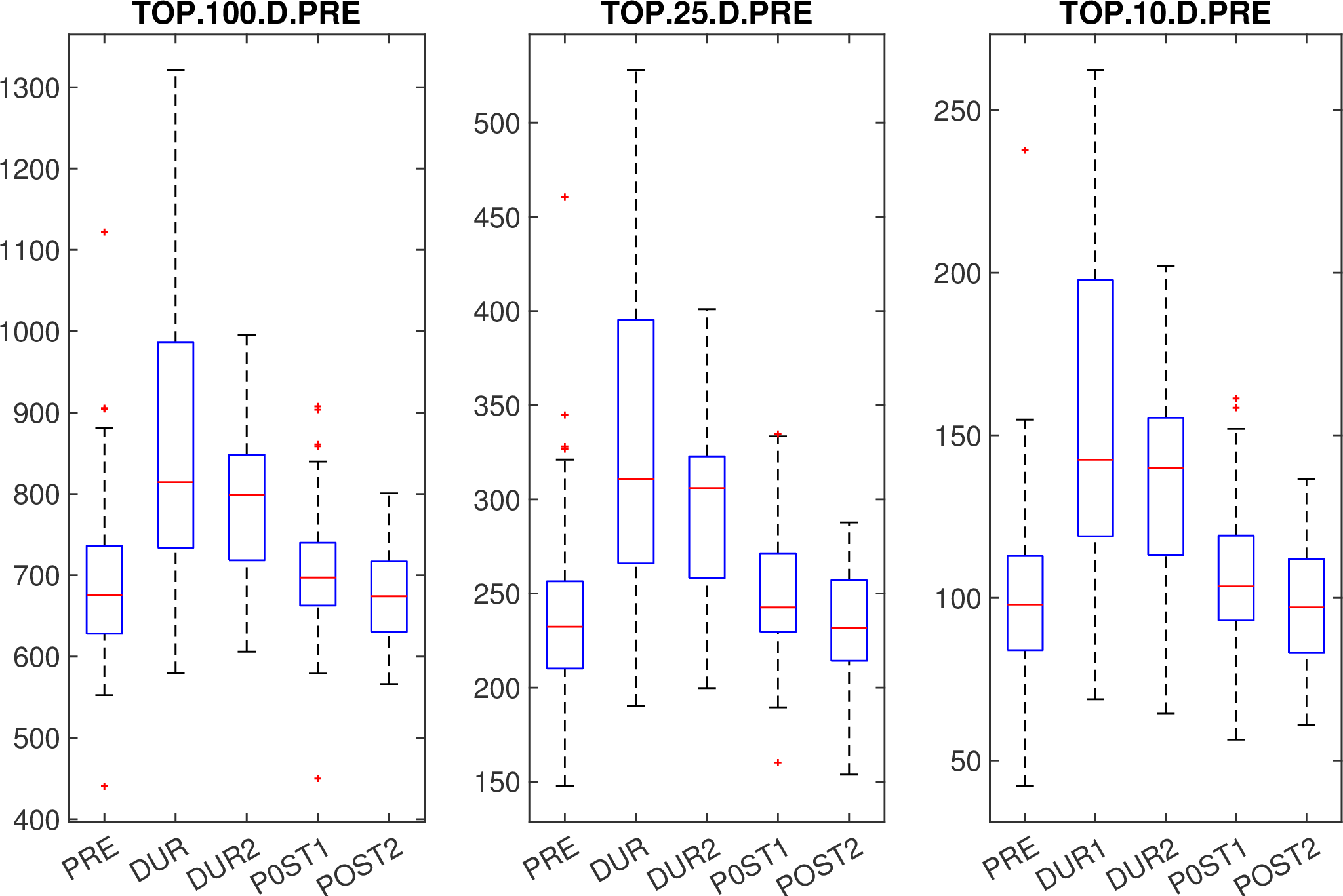

Supplement: S1 Fig — Distribution of scores for (a) Factor10, (b) Factor25, (c) Factor100 calculated respectively with the top 10, 25, and 100 genes differentially expressed in the longitudinal analysis of DURING1 vs. PRE. Median (red lines) and interquartile (blue boxes) of factor scores distributions are represented for the 5 longitudinal points PRE, DURING1, DURING2, POST1, and POST2. Figure is based on data in S6 Data. (TIF) [file pbio.3002089.s001.tif]

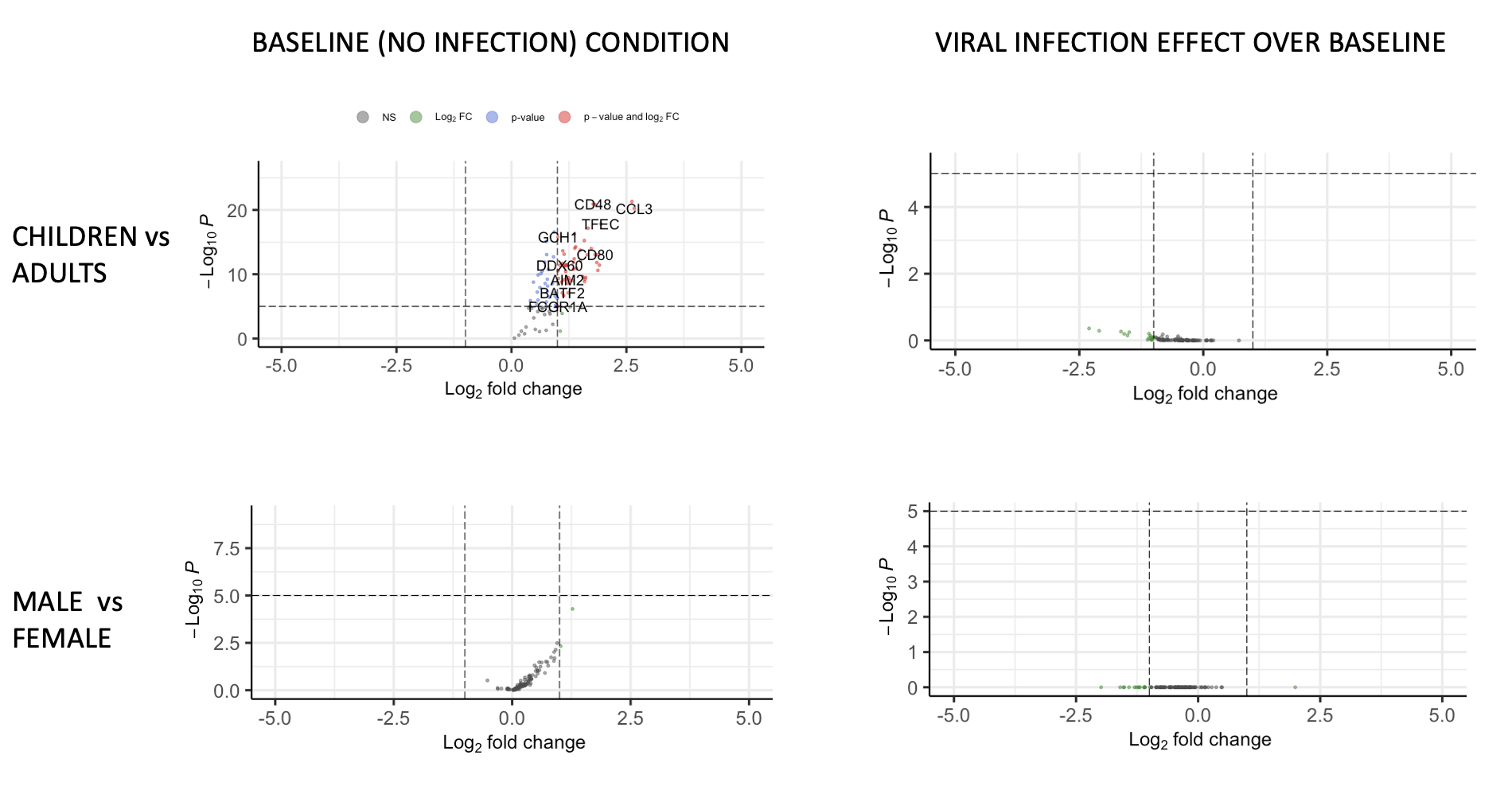

Supplement: S2 Fig — Comparison across age (children vs. adults) and sex groups (male vs. female) at baseline (i.e., in samples negative for respiratory viruses) and in positive samples (infection effect over baseline). Volcano plots were restricted to the top 100 immune genes overexpressed in the longitudinal analysis, used to build the metric F100. Figure is based on data in S7 Data. (TIF) [file pbio.3002089.s002.tif]

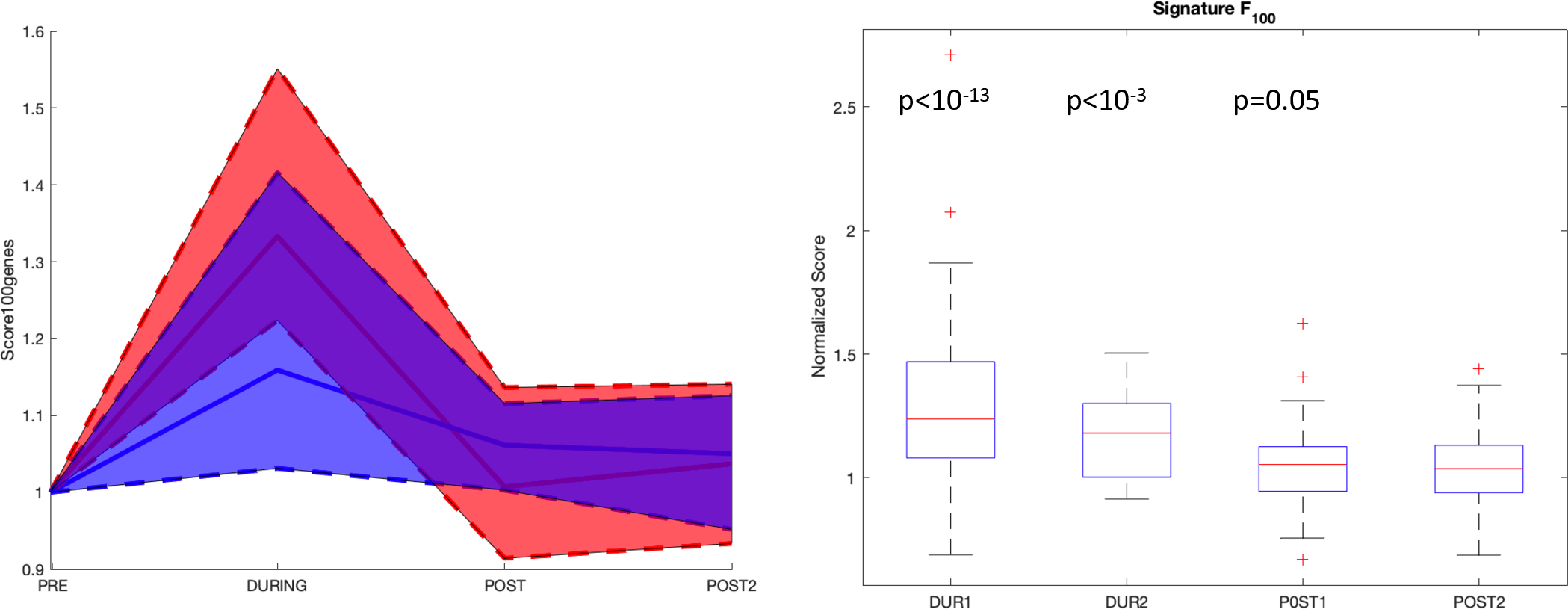

Supplement: S3 Fig — (a) Medians (straight lines) and interquartile (shaded areas) of scores based on F100 for longitudinal symptomatic (red) and asymptomatic (blue) episodes normalized by the respective PRE sample. (b) Scores F100 of longitudinal samples, calculated using the top 10 genes differentially expressed in the DURING1 vs. PRE comparison, normalized by the respective PRE sample in each episode. Red lines in boxplot are the median of distributions and blue boxes are the interquartile. Whiskers extend to all non-outliers of the distribution. Outliers are represented with red + symbol. For each group, we reported significant p-values of a Wilcoxon signed rank test for the hypothesis that the dataset comes from a distribution with median = 1 at the 5% significance level. Figure is based on data in S8 Data. (TIF) [file pbio.3002089.s003.tif]

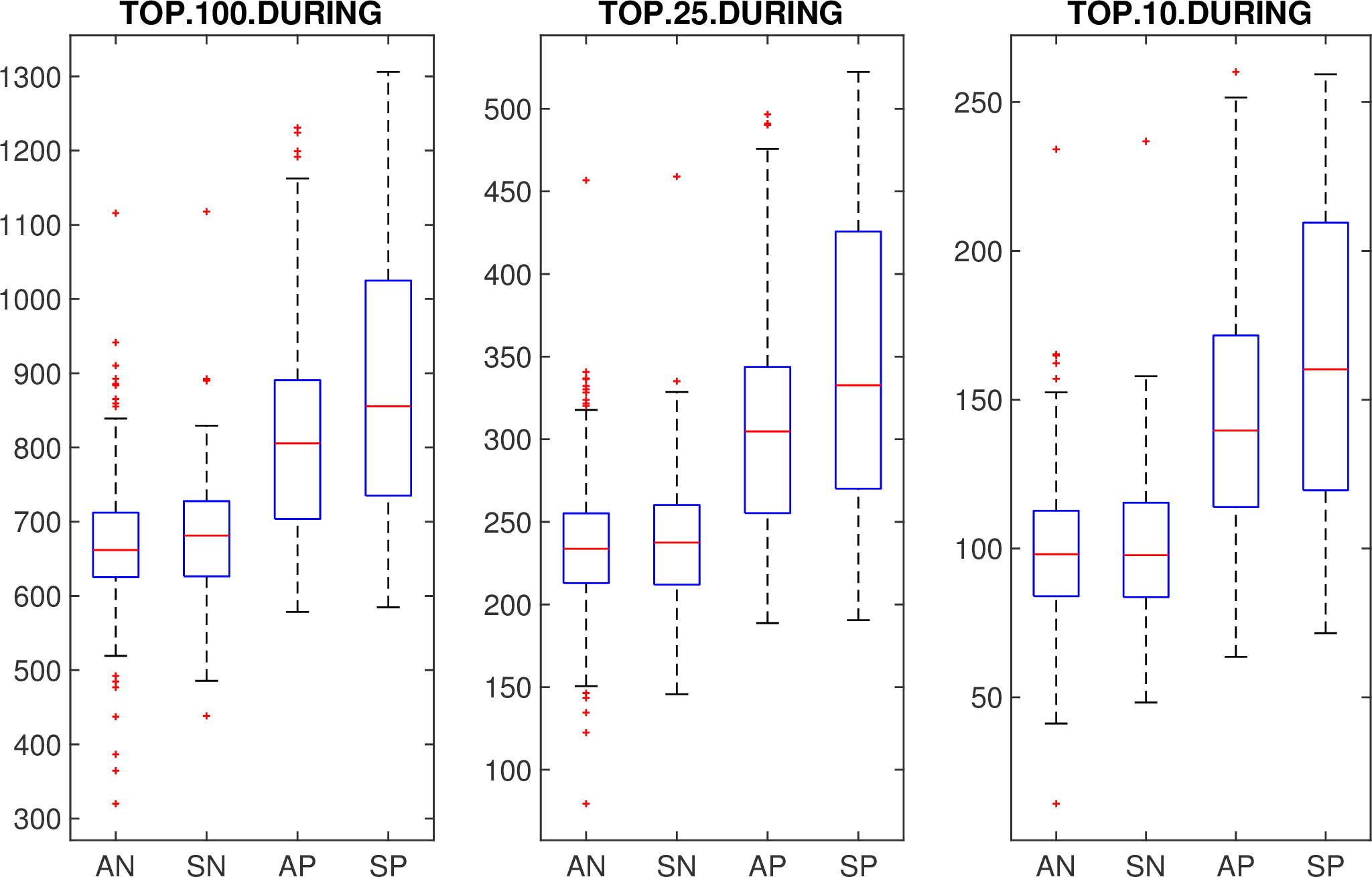

Supplement: S4 Fig — Distribution of scores for Factor10, Factor25, Factor100 calculated respectively with the top 10, 25, and 100 genes differentially expressed in the longitudinal analysis of DURING1 vs. PRE. Medians (red lines) and interquartile (blue boxes) of factor scores distributions are represented for the 4 groups of asymptomatic negative (AN), symptomatic negative (SN), asymptomatic positive (SP), and symptomatic positive (SP). Figure is based on data in S13 Data. (TIF) [file pbio.3002089.s004.tif]

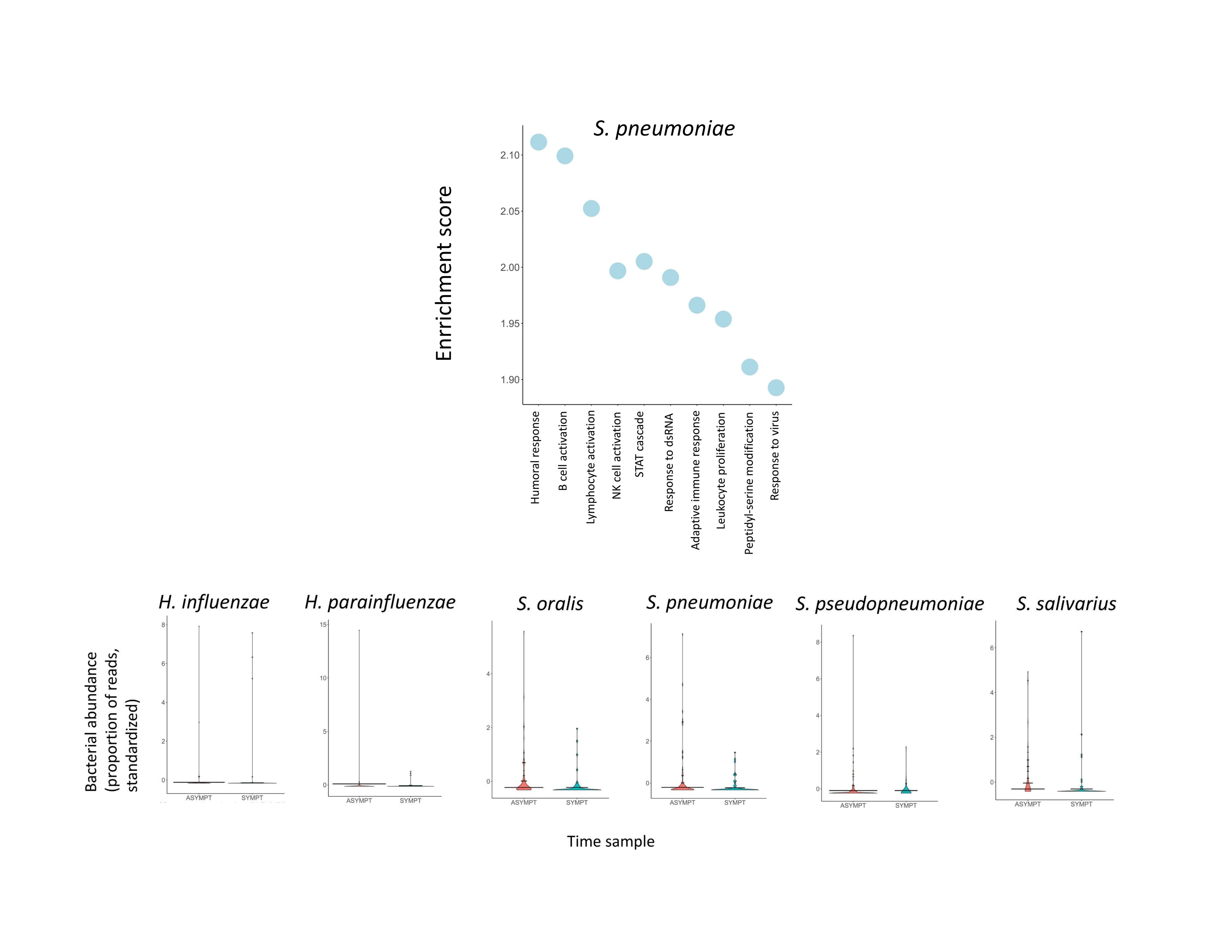

Supplement: S5 Fig — Panel (a): Top 10 significantly enriched biological processes, as found with GSEA (FDR < 0.05), derived from the analysis of transcriptomic interactions between abundance of bacteria and viral symptomatic viral infection for S. pneumoniae (only bacteria with significant association). Panel (b): Comparison of bacteria abundance in symptomatic vs. asymptomatic samples. Figure is based on data in S15 Data. (TIF) [file pbio.3002089.s005.tif]

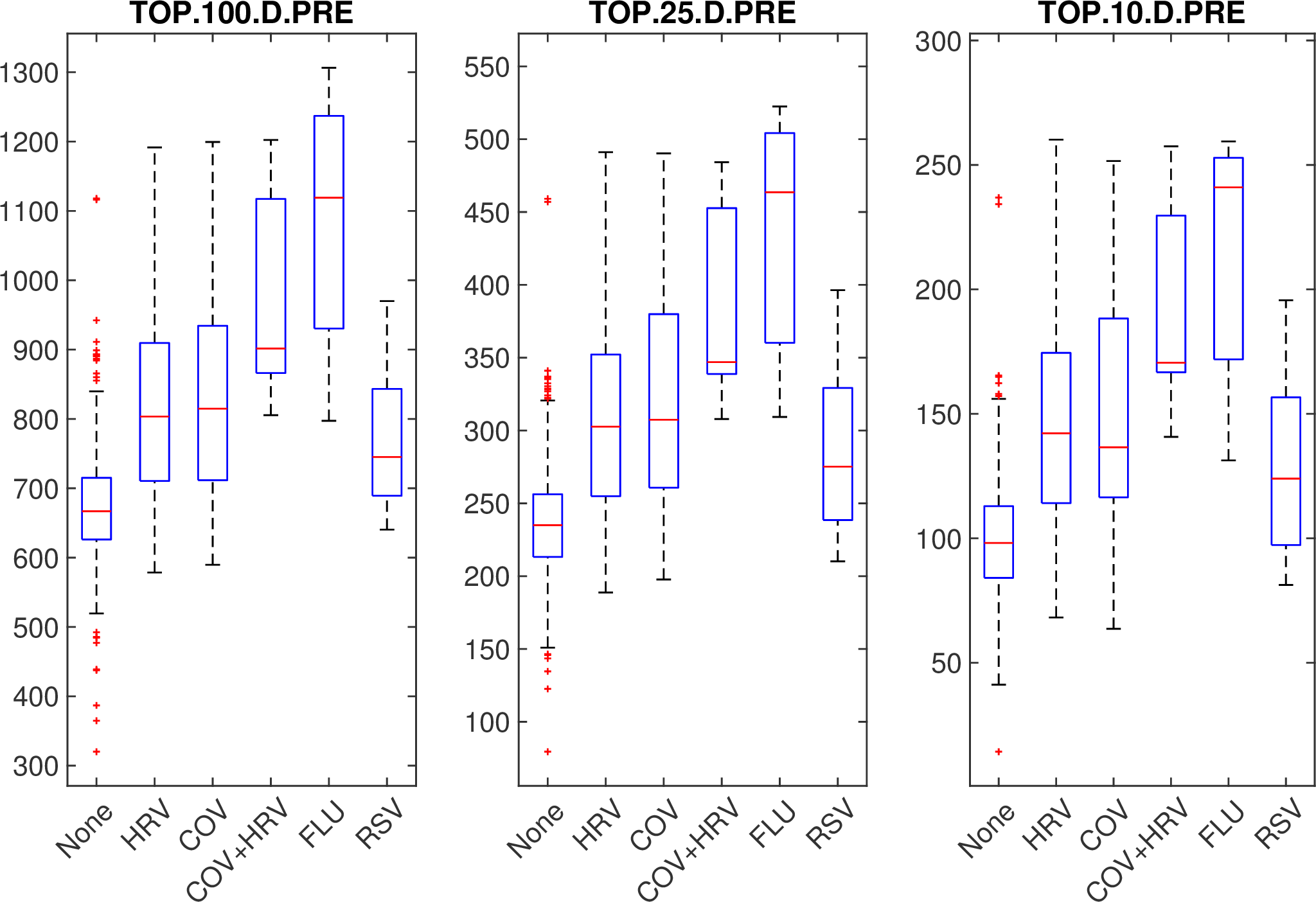

Supplement: S6 Fig — Distribution of scores for Factor10, Factor25, Factor100 calculated respectively with the top 10, 25, and 100 genes DE in the longitudinal analysis of DURING1 vs. PRE. Medians (red lines) and interquartile (blue boxes) of factor scores distributions are represented for plus negative baseline samples (None) and for the 5 viral infection types analyzed (rhinovirus (HRV), coronavirus (COV), coinfection of coronavirus and rhinovirus (COV+HRV), influenza (FLU), and respiratory syncytial virus (RSV)). Figure is based on data in S21 Data. (TIF) [file pbio.3002089.s006.tif]

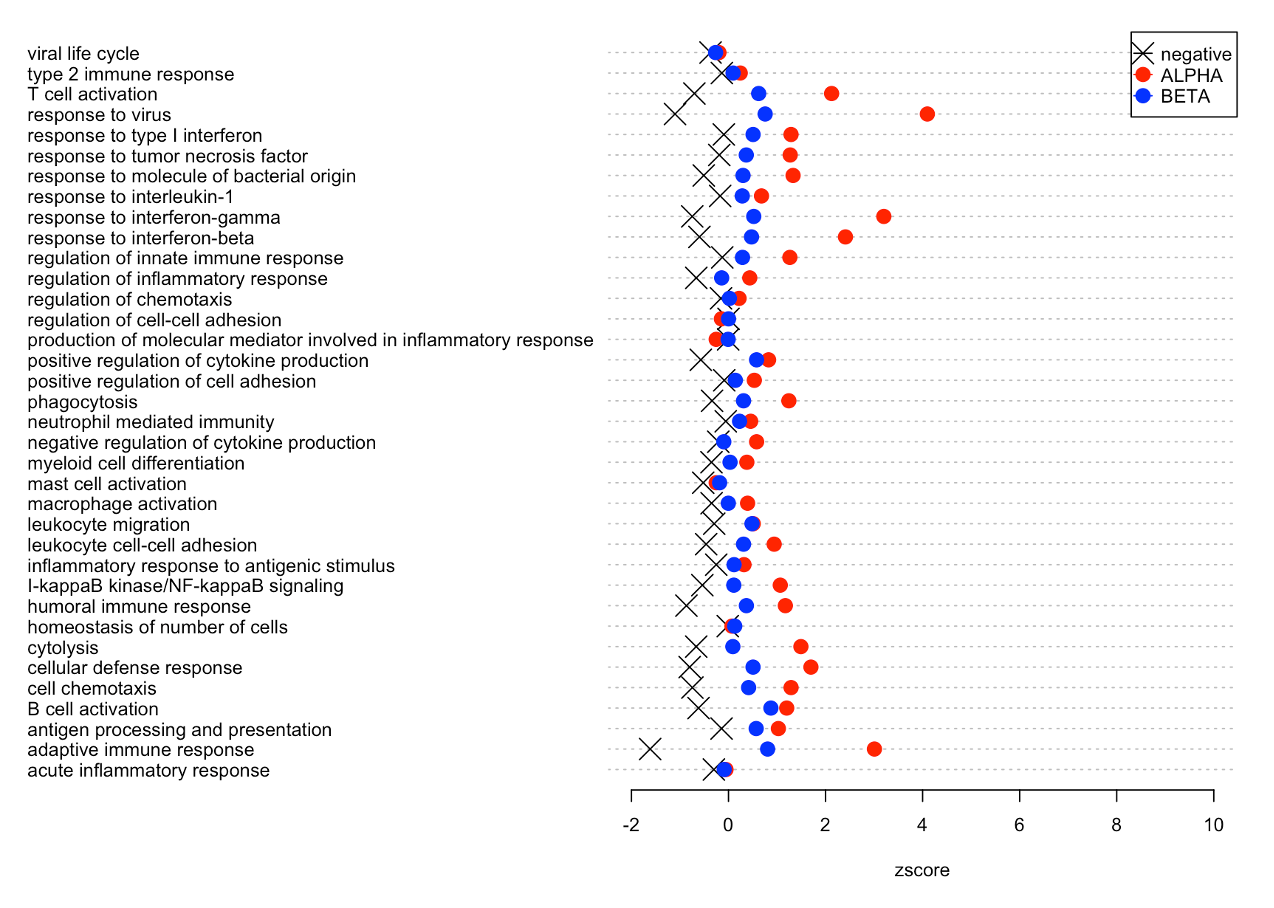

Supplement: S7 Fig — Comparison across the top immune-related biological processes enriched during infection (GSVA). Enrichment is quantified by the median zscore of samples that tested positive for each virus (colored dots) and compared to the median zscore across negative samples (black cross). Preselected processes are those overrepresented in the longitudinal and symptomatic analyses, as in Fig 6B. Figure is obtained applying GSVA to count data normalized with DESEQ (code and count data table in repository at https://github.com/RabadanLab/ViromeofManhattan). (TIF) [file pbio.3002089.s007.tif]

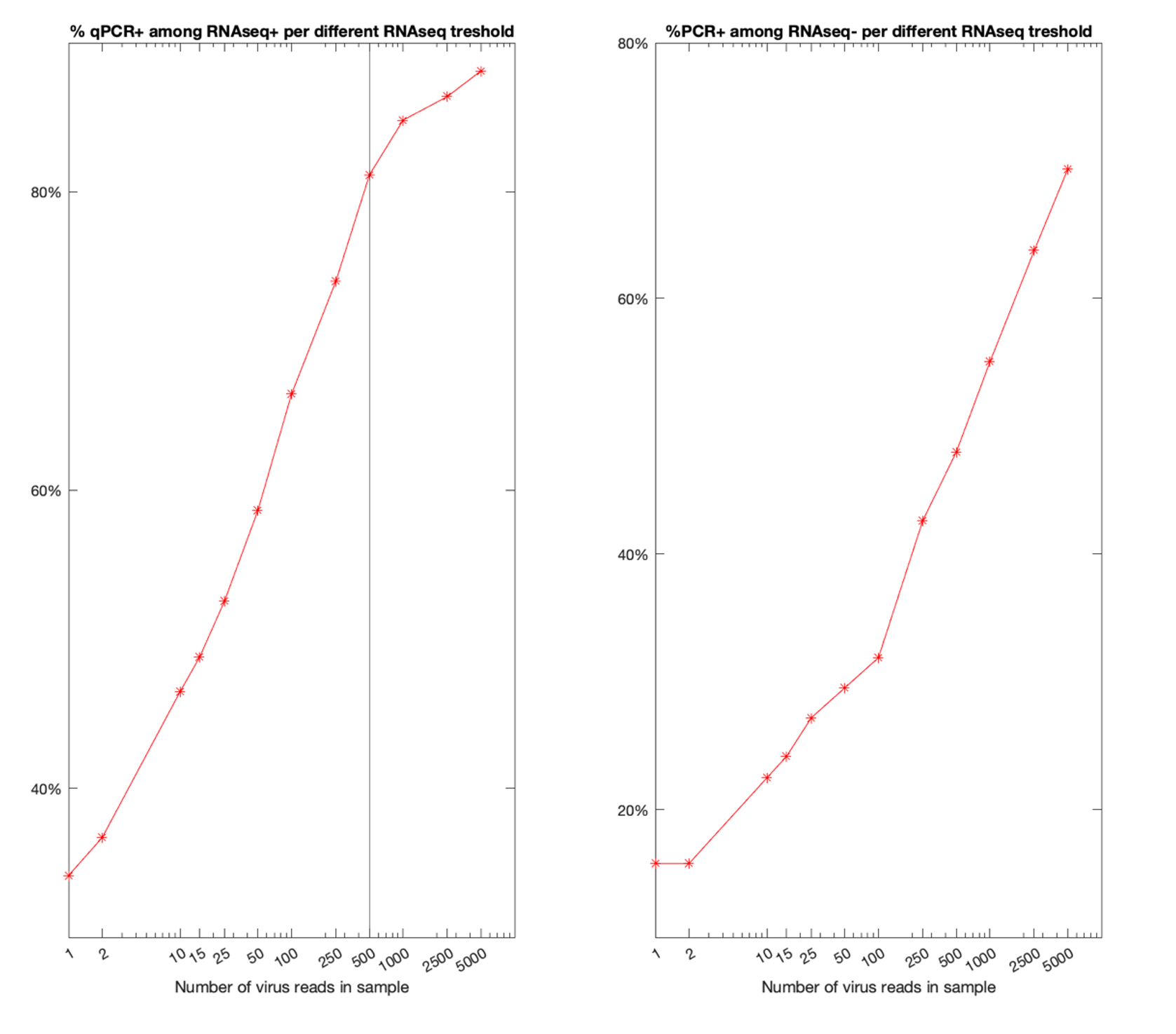

Supplement: S8 Fig — Only viruses included in the RVP are included in this comparison. First panel shows the percent of samples testing positive with qPCR, per increasing thresholds of RNAseq positivity (minimum number of reads of the virus identified by qPCR found in the sample). In formula, that is qPCR+(RNAseq>x)RNAseq>x. The second panel quantifies the percentage of PCR positivity below different threshold of RNAseq positivity. In formula, that is qPCR+(RNAseq<x)qPCR+. The vertical black line indicates the threshold chosen in our analysis. Figure is based on data in S23 Data. (TIF) [file pbio.3002089.s008.tif]

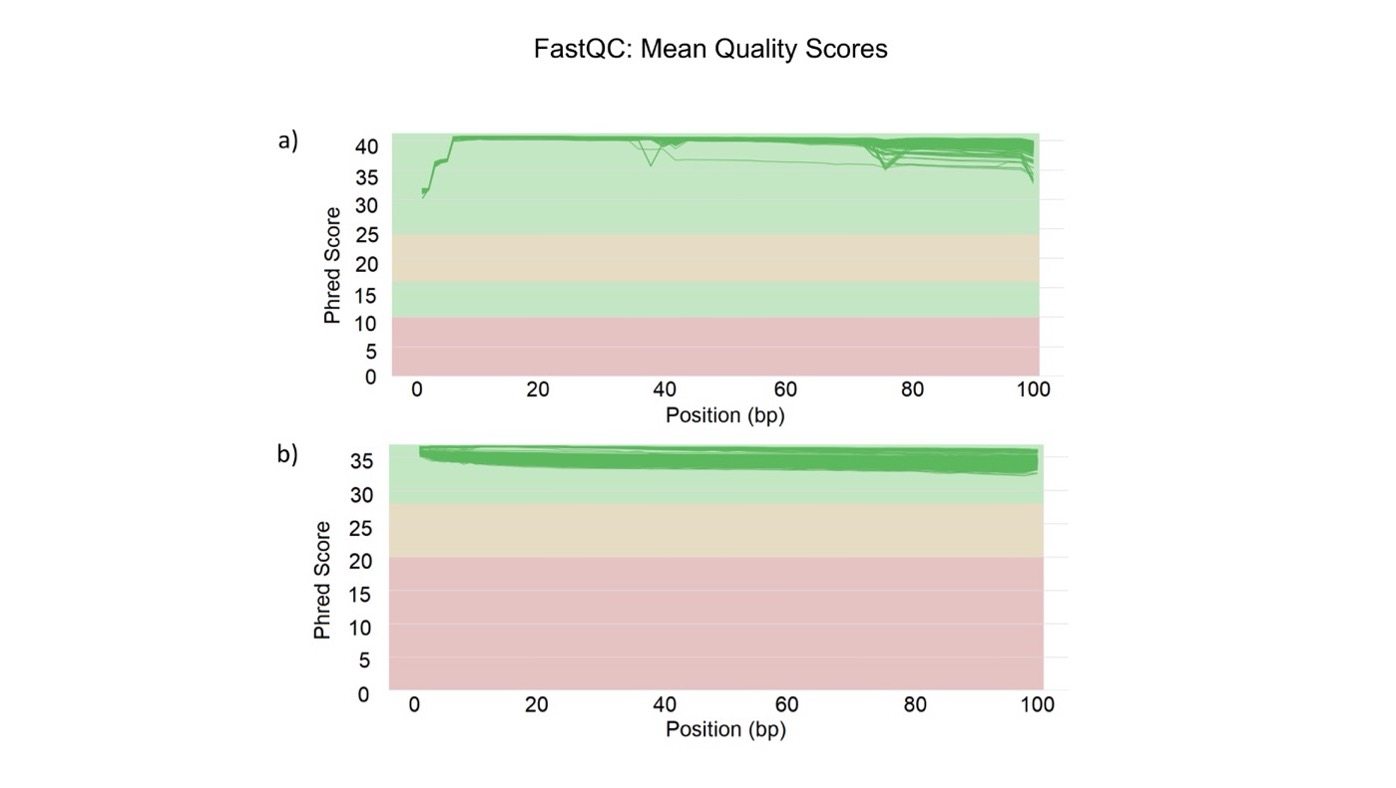

Supplement: S9 Fig — FASTQC summary plot of the reads quality for samples processed with single-end protocol and paired-end. Figure obtained by running FASTQC on raw files submitted on GEO repository with accession number GSE223679. (TIF) [file pbio.3002089.s009.tif]
