# Supplementary material for: Virome Data Explorer: A web resource to longitudinally explore respiratory viral infections, their interactions with other pathogens and host transcriptomic changes in over 100 people
Source: PLoS Biol. 2024 Jan 18;22(1):e3002089. doi: 10.1371/journal.pbio.3002089 (PMC10796020; doi:10.1371/journal.pbio.3002089)
Supplement: S22 Data — Age and sex of participants included in this study. (DOCX) [file pbio.3002089.s031.docx]

**Table S12**: **Cohort.** Age and Sex of participants included in this study.

|  | TOT | MEDICAL CENTER | DAYCARE_1 | DAYCARE_2 | HIGH SCHOOL | ER  DOCTORS |
| --- | --- | --- | --- | --- | --- | --- |
|  |  |  |  |  |  |  |
| PARTICIPANTS | 104 | 25 | 10 | 27 | 30 | 12 |
| ADULTS | 61 | 25 | 5 | 9 | 10 | 12 |
| TEENAGERS | 18 | 0 | 0 | 0 | 18 | 0 |
| CHILDREN | 23 | 0 | 5 | 18 | 0 | 0 |
| UNSPECIFIED | 2 | 0 | 0 | 0 | 2 | 0 |
|  |  |  |  |  |  |  |
| FEMALE | 58 | 14 | 6 | 17 | 13 | 8 |
| MALE | 46 | 11 | 4 | 10 | 17 | 4 |
|  |  |  |  |  |  |  |
